# Piloting a participatory, community-based health information system for strengthening community-based health services: findings of a cluster-randomized controlled trial in the slums of Freetown, Sierra Leone

**DOI:** 10.7189/jogh.09.010418

**Published:** 2019-06

**Authors:** Emily Cummings O’Connor, Jennifer Hutain, Megan Christensen, Musa Sahid Kamara, Abu Conteh, Eric Sarriot, Thomas T Samba, Henry B Perry

**Affiliations:** 1 Independent consultant, Freetown, Sierra Leone; 2 Concern Worldwide/Sierra Leone, Freetown, Sierra Leone; 3 Concern Worldwide/US, New York, New York, USA; 4 Formerly Concern Worldwide/Sierra Leone, Freetown, Sierra Leone; 5 Ministry of Health and Sanitation, Freetown, Sierra Leone; 6 Save the Children/US, Washington, D.C., USA; 7 Department of International Health, Johns Hopkins Bloomberg School of Public Health, Baltimore, Maryland, USA

## Abstract

**Background:**

Although community engagement has been promoted as a strategy for health systems strengthening, there is need for more evidence for effectiveness of this approach. We describe an operations research (OR) Study and assessment of one form of community engagement, the development and implementation of a participatory community-based health information system (PCBHIS), in slum communities in Freetown, Sierra Leone.

**Methods:**

A child survival project was implemented in 10 slum communities, which were then randomly allocated to intervention (PCBHIS) and comparison areas. In the 5 PCBHIS communities, the findings from monthly reports submitted by community health workers (CHWs) and verbal autopsy findings for deaths of children who died before reaching 5 years of age, were processed and shared at bimonthly meetings in each community. These meetings, called Community Health Data Review (CHDR) meetings, were attended by community leaders, including members of the Ward Development Committee (WDC) and Health Management Committee (HMC), by the CHW Peer Supervisors, and by representatives of the Peripheral Health Unit. Following a review of the information, attendees proposed actions to strengthen community-based health services in their community. These meetings were held over a period of 20 months from July 2015 to March 2017. At baseline and endline, knowledge, practice and coverage (KPC) surveys measured household health-related behaviors and care-seeking behaviors. The capacity of HMCs and WDCs to engage with the local health system was also measured at baseline and endline. Reports of CHW household contact and assessments of CHW quality were obtained in the endline KPC household survey, and household contacts measured in monthly submitted reports were also tabulated.

**Results:**

The self-assessment scores of WDCs’ capacity to fulfil their roles improved more in the intervention than in the comparison area for all six components, but for only 1 of the 6 was the improvement statistically significant (monthly and quarterly meetings in which Peer Supervisor and/or CHW supervision was an agenda item). The scores for the HMCs improved *less* in the intervention area than in the comparison area for all six components, but none of these differences were statistically significant. Topics of discussion in CHDRs focused primarily on CHW functionality. All three indicators of CHW functioning (as measured by reports submitted from CHWs) improved more in the intervention area relative to the comparison area, with 2 out of 3 measures of improvement reaching statistical significance. Five of 7 household behaviors judged to be amenable to promotion by CHWs improved more in the intervention area than in the comparison area, and 2 out of the 5 were statistically significant (feeding colostrum and appropriate infant and young child feeding). Four of the 6 care-seeking behaviors judged to be amenable to promotion by CHWs improved more in the intervention area than in the comparison area, and 1 was statistically significant (treatment of diarrhea with ORS and zinc). None of the findings that favored the comparison area were statistically significant.

**Conclusions:**

This study was implemented in challenging circumstances. The OR Study intervention was delayed because of interruptions in finalizing the national CHW policy, two separate cholera epidemics, and the Ebola epidemic lasting more than 2 years. Weaknesses in the CHW intervention severely limited the extent to which the PCBHIS could be used to observe trends in mortality and morbidity. Nonetheless, the positive results achieved in the area of functionality of the CHW intervention and community structure capacity are encouraging. Results suggest there is value in further methodologically rigorous investigations into improving community-based health system functioning through a similar approach to community engagement.

As practitioners and policy makers seek to accelerate the decline in maternal, neonatal and child mortality and advance the achievement of the Sustainable Development Goals and universal access to health care, there has been growing interest and investment in health systems strengthening [1]. Concurrently, there is growing evidence that community engagement has the potential to improve the impact of health interventions, facilitate responses to public health emergencies and disasters, and strengthen health systems as a whole [2-4].

Community Health Workers (CHWs) can serve as the link between the community and the health system and offer an increasingly important resource for assisting health systems to meet the needs of communities [5,6]. Through provision of household-level preventive, promotive, and treatment services, recent estimates state that expanding access to CHWs could prevent 2.6 million deaths of mothers and children each year [7]. However, challenges remain to building strong CHW programs that deliver high-quality evidence-based interventions and achieve high levels of population coverage.

In addition, the documented experiences and evidence of effectiveness of engaging communities to strengthen health systems remain limited [2]. Sacks et al [2] highlight the critical nexus of health system strengthening and community-oriented programming and call for the inclusion of the community as a key actor in the process of health systems strengthening.

Momentum around community engagement in health systems may be building. In 2017, the World Health Organization released a framework highlighting the role of community engagement for quality, people-centered, and resilient health services. However, a 2016 review concluded that community engagement will only strengthen health systems if community capacity is first strengthened [8].

This paper reports the findings of an operations research (OR) study in which a participatory, community-based health information system (PCBHIS) was implemented in slum communities in Freetown, Sierra Leone. The study was undertaken to assess the effect of a PCBHIS on the ability of community structures to take actions in response to locally generated data and to improve community-level health outcomes.

## Study setting

**Figure 1:**
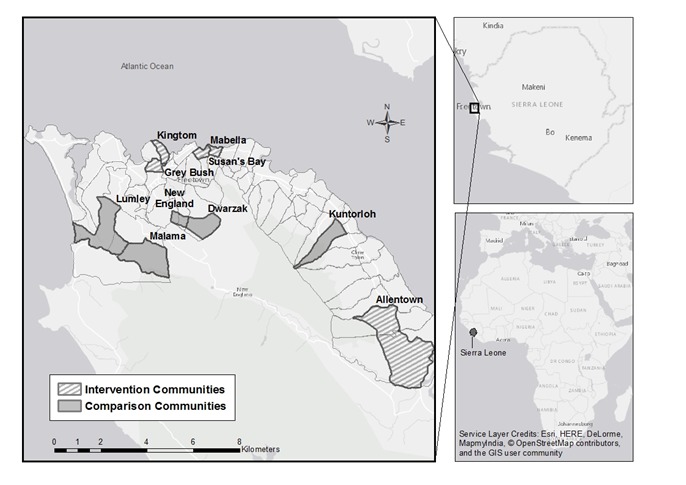
Map of Freetown showing intervention and comparison communities.

The study was implemented in 10 non-contiguous urban communities of Freetown, Western Urban District, Sierra Leone (**Table 1** and **Figure 1**). The population of under-5 children, pregnant women, and mothers of under-5 children in these communities was determined through a participatory community-based census conducted in 2012-13. The total population of the study area in 2008 according to the national census was 166 417. The communities in the study area are primarily unplanned, informal settlements characterized by overcrowding, low-quality housing, limited access to clean water, and lack of sanitation. Flooding is common in parts of the study area, causing fatalities every year. The entire city of Freetown is densely populated, with an estimated 1.1 million inhabitants within an area of 138 square miles [9].

**Table 1:** Operations research study population by intervention and comparison communities

Community	Number of pregnant women*	Number of mothers with a child younger than 5 years of age*	Number of children younger than 5 years of age*	Total number of beneficiaries (mothers and young children)
**Intervention area**				
Allentown	639	3868	5467	9974
Mabella	299	1640	2195	4134
Susan’s Bay	298	1481	2201	3881
Grey Bush	132	907	1191	2230
Kingtom	164	1167	1561	2892
Total	1532	9063	12 615	23 111
**Comparison area**				
Kuntorloh	638	4100	5824	10 562
Dwarzack	530	3306	4606	8442
New England	322	1958	2593	4873
Lumley	395	2467	3448	6310
Malama	604	3598	5056	9258
Total	2489	15 429	21 527	39 445

*Determined through a participatory, community-based census conducted in 2012 and 2013.

The PCBHIS intervention was carried out in five communities that made up the intervention area. The remaining five communities served as the comparison area where the PCBHIS intervention was not implemented. Concern Worldwide, an international non-governmental organization, worked with the Ministry of Health and Sanitation (MOHS), the District Health Management Team (DHMT), the Freetown City Council (FCC), and local communities to implement a child survival project (CSP), as discussed below, as well as the OR Study.

The 2016 Human Development Index ranked Sierra Leone 179^th^ out of 188 countries [10]. While health outcomes have improved somewhat since a 10-year civil war ended in 2002, the country continues to be challenged by shocks: cholera epidemics in 2012 and 2013 followed by an Ebola epidemic that spanned more than two years (2014-2016). The Western Area Urban District, which includes Freetown and the study area, was one of the worst affected areas during all of these public health crises.

National health indices remain among the worst in the world. According to recent estimates by UNICEF, the maternal mortality ratio is the highest in the world (1360 maternal deaths per 100 000 live births), and the under-5 mortality rate is the fourth highest in the world (114 under-5 deaths per 1000 live births [11].

## Local health system structure

Government primary health care facilities available at the sub-district level are referred to as Peripheral Health Units (PHUs). The DHMT plans, coordinates and supervises services at PHUs as well as interventions in the community such as public health campaigns. The FCC also plays a key supporting role in the delivery of community health services.

Each PHU is supported by a Health Management Committee (HMC). The HMC serves as a liaison between the PHU and the community and supports community mobilization and outreach activities from the PHU. Each of the 39 wards within the Freetown municipal area has an elected Ward Development Committee (WDC), responsible for engaging community members on general development activities. The WDC chair sits as a Councilor on the FCC. Each community in the study area covers 1-2 wards. In cases where the community covers more than one ward, both WDCs participated in the study. Roles and methods for HMCs and WDCs to act as the liaison to the community have not been well-developed. While the Government of Sierra Leone highlights the need for community engagement in recently developed policy documents, such as the Basic Package of Essential Health Services (BPEHS) 2015-2020, it focuses on CHWs to fulfil this role. The BPEHS recognizes HMCs and WDCs as community actors but does not outline roles or the ways in which they should fit into the health system [12].

## The national community health worker program

The MOHS finalised a national community health worker (CHW) policy in 2012 and began operationalising it in early 2014. With the Ebola outbreak also emerging in 2014, the rollout of the CHW policy and the extent of government ownership of the CHW program were impaired. During the period of the OR study, Concern Worldwide supported the introduction of a CHW program across the full study area in line with the 2012 CHW policy, tailored to the urban context and approved by the Western Area DHMT. These CHWs were involved only in health promotion, referral, and surveillance; they did not provide any curative treatments. Under the newly developed National CHW Policy 2016-2020, community structures are to have a role in reviewing community-level health data, but no mechanism for this review has yet been determined [13].

## The Child Survival Project

The OR Study described below was embedded within a USAID-supported child survival project (CSP), known locally as *Al Pikin Fo Liv* (All Children Should Live), with co-funding from Irish Aid. The CSP was implemented in all of the 10 study communities listed in Table 1 from October 2011 to June 2017 (see also **Figure 1**).

1325 volunteer CHWs were trained by Concern Worldwide CSP staff and the Western Area DHMT using the Sierra Leone MOHS 2012 National CHW Program training materials. Introductory sessions on the CHW Program were held for community leaders by CSP staff and the Western Area DHMT. 606 male and 709 female community members were then selected by community leaders to be trained as CHWs as per National CHW Program selection criteria. About two-thirds of those trained were between 18 and 34 years of age, and 21% were 35-54 years old. Only 4% of CHWs were over 55 years of age. Almost 60% of those trained had completed some secondary school and 5% classified themselves as non-literate. The number of CHWs trained was calculated using a CSP- and community-led census and policy-mandated population-to-CHW ratios. 106 CHWs were selected as Peer Supervisors by CSP together with community leaders, based on their performance during initial CHW training. Peer Supervisors were given additional training by the CSP, and assigned 8-12 CHWs to supervise. These Peer Supervisors were attached to the specific area, or “zone” of the community where their supervisees operated. At least one HMC and one WDC member from the same zone provided oversight and assistance to the Peer Supervisor. Using the participatory census results, CHWs were assigned 25 households, generally in close proximity to their own homes, to visit monthly and disseminate health messages, check for danger signs of illness, and collect vital event and morbidity data using MOHS registers.

**Figure 2:**
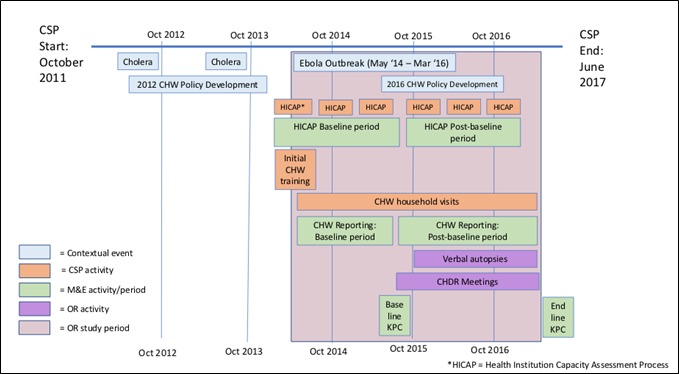
Timeline of key contextual events and key activities of the child survival project, the operations research study, and data collection.

The CHW intervention was designed so that every household with a pregnant woman or under-5 child in the 10 study communities would receive a visit at least once per month. CHWs made home visits and collected household-level data for 13 months (May 2014 – June 2015) before the OR intervention (the PCBHIS) began in July 2015. The CHW intervention and OR activities then ran concurrently for a subsequent 20 months (July 2015 – March 2017). See **Figure 2** for a timeline of the CSP and the OR Study. Appendix S1 Table S1 in **Online Supplementary Document** contains the objectives and activities of the CSP.

## THE OPERATIONS RESEARCH STUDY: DESIGN AND METHODS

### Study design

The OR Study was developed in 2011 and updated in 2012 and 2013 through a collaborative process between the Johns Hopkins University and Concern Worldwide. The objectives of the study were 1) to assess the extent to which the PCBHIS facilitated local community structures to use data to plan and implement actions for improving maternal, neonatal, and child health (MNCH) and 2) to assess the extent to which this contributed to improved community-level MNCH outcomes. The study was a cluster-randomized controlled trial, with communities randomly selected to either the intervention or comparison area. The OR Study intervention consisted of two activities in addition to the CSP activities:

1. Implementation of meetings every two months to support HMCs, WDCs, and Peer Supervisors to review household-level data collected by CHWs and determine actions in response to this data. These meetings are referred to as Community Health Data Review (CHDR) meetings.
2. Verbal autopsies (VAs) for deaths of under-5 children which had been registered by CHWs. This activity is described in detail in a companion paper [14].

**Table 2** summarizes the activities that took place in the intervention and comparison areas.

**Table 2:** Intervention vs comparison area activities

Activity	Intervention communities	Comparison communities
**Child survival project activities:**		
Recruit and train CHWs and Peer Supervisors (a one-time activity)	Yes	Yes
Support CHWs to conduct home visits to check for danger signs, make referrals to the PHU, and deliver behavior change messages (ongoing)	Yes	Yes
Implement the Health Institution Capacity Assessment Process (every six months)	Yes	Yes
**Operations research activities:**		
Process monthly CHW reports from the intervention communities (monthly)	Yes	**No**
Obtain verbal autopsies from caretakers for deaths of children who died before reaching 5 y of age in the intervention communities (ongoing)	Yes	**No**
Facilitate the holding of bimonthly Community Health Data Review Meetings with Peer Supervisors, CSP staff, and PHU staff to share local surveillance data and verbal autopsy information in the intervention communities (every two months)	Yes	**No**
**Child survival project monitoring activities:**		
Collect CHW reports (monthly)	Yes	Yes
Collect Health Institution Capacity Assessment Process (HICAP) scores (every six months)	Yes	Yes
**Evaluation activities:**		
Conduct household knowledge, practice, and coverage surveys (at baseline and endline)	Yes	Yes

The source of the data for the PCBHIS was the monthly CHW reports. The original study design, developed in 2011, called for CHWs to collect morbidity and mortality data only in the intervention area, using data collection materials designed by the study through its formative research. However, the MOHS Policy for Community Health Workers in 2012 required that morbidity and mortality data be collected by CHWs nationally and thus was collected across the entire study area, using standardized MOHS materials. As a result, the initially envisioned OR Study intervention had to be altered, and the OR Study was unable to use its own forms for registering morbidity and mortality in the intervention area communities. Numerous other challenges arose that required modification of the original study design. These are described in Table S1 and S2 Appendix S2 in **Online Supplementary Document**.

### The principal operations research study intervention: Community Health Data Review Meetings

CHDR Meetings were designed to be held in each of the five intervention communities every two months during the period of OR implementation. The purpose of these meetings was to support the HMC, WDC, PHU staff, and CHW Peer Supervisors of that community to engage with health data that was collected by the CHWs in that specific intervention community, and determine actions in response. All HMC members, WDC members, and Peer Supervisors for the community and the PHU In-charge were invited to each meeting.

Each community had 5-12 CHW Peer Supervisors depending on the community population. All communities had one HMC consisting of 15 members. Photos of CHDR meetings are included in Appendix S3 in **Online Supplementary Document**.

Generally, data for the preceding 4-6 months were reviewed in each CHDR meeting. The HMC Chair for the community chaired the meeting. Sessions were facilitated by OR Study staff, but, over time, participants increasingly took the lead in reviewing the data and reporting the findings. OR Study staff analyzed CHW-collected data with the CSP staff attached to that community prior to the meeting and determined topics and data to present in CHDRs. OR Study staff prepared simple data sheets to be used by participants, and participants used them to draw and interpret bar charts in front of the group, as shown in in Appendix S3 in **Online Supplementary Document**, Figures S1 and S2. Records were kept of discussion topics.

Six months after the initiation of CHDR meetings, OR staff began to share information from verbal autopsies on deaths of children who died before reaching five years of age in the meetings. The discussion focused on the caretaker’s account of the events leading up to the death of the child and the efforts undertaken by the family to obtain treatment. A companion paper [14] describes the community-collaborative verbal autopsy process and the results obtained.

Following the review of data, CHDR participants developed action points as seen in Appendix S3 Figure S2 in **Online Supplementary Document**. Action points were documented during the meeting on flipchart paper which the HMC Chairman kept after the meeting. Action points from previous meetings were reviewed in subsequent meetings, and discussions held on the extent to which actions had been completed.

### Indicators of the capacity of community committees to engage with the local health system: the Health Institution Capacity Assessment Process (HICAP)

The capacity of the community to engage with the local health system was assessed with the Health Institution Capacity Assessment Process (HICAP). This is a participatory, self-assessment tool used to assess local organizational capacity to perform their functions. The tool and the process for using it were developed by Concern Worldwide and tailored for use by the HMCs and WDCs in the study area (see Appendix S5 in **Online Supplementary Document** for a copy of this instrument). HMCs and WDCs used consensus decision-making to determine their structures’ score for 21 indicators. Each indicator has five pre-determined criteria and associated values. Six HICAP assessments were conducted with each of the ten HMCs and ten WDCs over a 37-month period, taking place at intervals of 6-9 months. Assessments were conducted through day-long workshops in which the HMCs and WDCs came together in each community. Each HMC and WDC determined its own score.

Following each assessment, indicator values were recorded and calculated as percentages, representing scores out of a maximum score of 5. For analysis purposes, indicator values determined in the three assessments that took place during the baseline period were averaged and considered as baseline values, and those scores determined from assessments following this period were considered as post-baseline values (see **Figure 2** for timing of the baseline and post-baseline periods). Further analyses were then conducted to determine the difference in differences between the intervention and comparison areas from the baseline to the post-baseline periods.

### Indicators of effective health system functioning: functionality of the CHW program as determined by rate of CHW reporting

Over a 34-month period, CHWs submitted monthly registers to their Peer Supervisor, and Peer Supervisors submitted monthly summary forms to the CSP and OR Study staffs. This information was summarized for each community and entered into an Excel database. Included were the number of CHWs and Peer Supervisors submitting reports and the number of households reached by CHWs, as reported by the CHWs in their own reports.

The 13 months preceding the initiation of CHDRs were considered as the baseline period, and baseline averages of each indicator were calculated for the intervention and comparison areas. The 21-month period following the initiation of CHDRs was considered the post-baseline period. As with baseline calculations, averages of each indicator were calculated for intervention and comparison areas during the post-baseline period. A difference-in- differences analysis was then conducted between the intervention and comparison areas from baseline to post-baseline.

### Indicators of effective health system functioning: health system utilization and household behaviors as determined from household surveys

A baseline knowledge, practice and coverage (KPC) survey collected data from both intervention and comparison areas in May 2015 (the month prior to initiation of the OR intervention). See Appendix S4, Tables S3 and S4 **Online Supplementary Document** for indicators measured. An endline survey measured the same indicators following the end of the OR intervention in April 2017. The survey used a structured questionnaire with a core module directed at mothers of children 0-23 months of age as well as a sub-module directed at mothers of children 0-23 months who had had symptoms of diarrhea, pneumonia or malaria within the two weeks prior to the survey. At endline, an additional module was added on frequency and quality of CHW home visits. See Appendix S4, Table S1 in **Online Supplementary Document** for indicators. 0 clusters in the intervention and comparison areas each were selected at random using a probability proportional to size methodology based on population projections from the most recent Government of Sierra Leone census. Ten interviews were conducted in each cluster through a random selection of households and a random selection of the respondent within the household. The baseline KPC survey had 299 respondents in the intervention area and 300 in the comparison area. The endline KPC survey had 379 respondents in the intervention area and 413 in the comparison area. The sampling methodology provided a 95% confidence interval of ±8% or less for the prevalence of indicators measured in the population.

Data were collected using digital data collection devices and uploaded to an electronic database. A difference-in-differences (DID) analysis was carried out to assess the degree to which changes in the intervention area were different from those in the comparison area. Results from the KPC survey concerning CHW visitation, obtained only at the time of the endline survey, were compared between the intervention and comparison areas.

The statistical significance of differences in simple comparisons between baseline and endline values of indicators or between the intervention and comparison areas were calculated using WINPEPI version 11.65 (Brixton Health, London, UK). The statistical significance of the DID estimate was assessed using a z-test based on the variances of its four component proportions.

### Ethical review

The OR Study protocol was approved by the Sierra Leone Ethics and Scientific Review Committee and was declared exempt from human subjects review by the Johns Hopkins University School of Public Health Ethical Review Board prior to study implementation. An Operations Research Steering Committee was established that consisted of Dr Perry as chairperson, national and local government representatives, UNICEF representatives, and Concern Worldwide representatives.

## RESULTS

### Community Health Data Review Meetings

Twenty-nine meetings were held over a 20-month period from July 2015 to April 2017. Each community in the intervention area was involved in five to seven meetings, and one community held an initial pilot meeting. The number of participants in meetings ranged from approximately 30 to 50 people. Generally, the same HMC members, WDC members, and Peer Supervisors attended each meeting. PHU In-Charges rarely attended meetings, but generally sent the same representative to each meeting. PHU staff attendance was not strong at the beginning of the process, but HMC members engaged PHU staff and attendance improved.

All meetings included a review and discussion of the number of CHWs reporting and number of Peer Supervisors reporting. Almost all meetings included a review of the number of households reached by CHWs during previous months, whether as an overall number compared to the target or as the average households reached per CHW compared to their monthly target of 25. Additional data that were reviewed and discussed, in approximate order of frequency, are shown in **Table 3**. All data presented were specific to the community except for the data from the two communities of Grey Bush and Kingtom since they were engaged in joint meetings.

**Table 3:** Frequency of data presented during Community Health Data Review Meetings

Data reported	Frequency of inclusion out of 29 CHDRs (%)
Number of CHWs reporting compared to total number of CHWs trained	29 (100%)
Number of Peer Supervisors reporting compared to number of total number of Peer Supervisors trained	29 (100%)
Number of households reached (or average number of households reached per CHW) as compared to targets	25 (86%)
Number of births reported by CHWs	22 (76%)
Number of deaths reported by CHWs	22 (76%)
Verbal autopsy results (case studies describing events leading to the death and themes emerging)	20 (69%)
Verbal autopsy results: physician-determined cause of death	10 (34%)
Number of illness symptoms reported by CHWs (diarrhea, fever, and/or cough)	10 (34%)
Number of CHW referrals, as reported by CHWs	5 (17%)
Number of CHW visits to pregnant women, as reported by CHWs	3 (10%)
Data from the Peripheral Health Unit (number of antenatal care visits and number of visits of sick children)	3 (10%)

CHDR – community health data review, CHW – community health worker

Discussions of data on CHW and Peer Supervisor reporting rates and on coverage of the community by CHWs centered on:

- Ways in which the HMC and WDC can motivate CHWs and Peer Supervisors
- The role of Peer Supervisors in supervising and motivating CHWs, particularly focusing on strategies for motivating the CHWs to reach more households and report data (including vital events) consistently and accurately
- Roles of CHWs, their target of visiting 25 households per month, and recording data accurately
- The effect of DHMT and MOHS activities (such as health campaigns, a mapping exercise to count all CHWs in the country, and selection of CHWs to be trained under a new national CHW Policy to be launched in 2017) on CHW motivation and functionality.

As detailed in **Table 3**, the data presented at the CHDR meetings and during the ensuing discussions focused overwhelmingly on the reporting rates of CHWs and the coverage of homes with visits from CHWs. It became increasingly clear that there was substantial under-reporting of morbidity data and vital events, even among those CHWs who were submitting monthly reports. The low level of completeness and quality of morbidity and mortality data led the OR study staff to focus discussions on data quality and on actions to improve quality and completeness, rather than on a review of health trends. While the OR study design had intended for CHDRs to focus on health trends and actions to address these trends, the data quality issues needed to be resolved first. As the CHDR meeting activity proceeded, the OR Study staff gradually began presenting data at a more granular level, often through group work between the Peer Supervisor, HMC members and WDC members covering a specific zone of the community.

For example, this small group would discuss each CHW who was responsible for working in their specific area and agree on strategies for motivating those that were not covering all households or were not reporting. This adjustment reduced the abstractness of the information, increased accountability, and enabled participants to have more practical discussions about data quality. CHW motivation as well as quality and completeness of CHW monthly reports were the most frequent topics of discussion in response to CHW-gathered data. A frequently agreed action was for community leaders to hold group or individual meetings with low-performing CHWs, or between CHWs and their Peer Supervisor to resolve disputes which had arisen. CHWs and Peer Supervisors expressed that simple recognition by community leaders was a strong motivating factor.

One Peer Supervisor said:

“At first the HMC and WDC see the CHW workers as something that is not important, but the data review meetings showed that the CHWs are working a lot. We have a very good relationship with the stakeholders after the data review meeting.”

Another Peer Supervisor said:

“CHDR meetings help me to know the lapses of my CHWs. After the comments during the meeting I am able to talk to my CHWs and encourage them to report.”

It is particularly interesting to note that the Peer Supervisor felt more able to review his own CHWs’ data during the CHDRs, and perhaps did not feel able to do this on his/her own. Project staff also expressed that the CHDRs assisted them in their own work by helping them to identify problems with the CHW data.

Presentations of verbal autopsy data generated active discussions about household level behaviors and health system capabilities. For example, participants sought clarity from PHU staff on clinic hours of operation and actions to take if no staff are found at the PHU. While community leaders may have been somewhat aware of some issues arising in CHDR meetings, the meetings gave them an opportunity to see them more concretely and to discuss possible solutions. Since community leaders face many competing priorities, including their own livelihoods, without this forum it is unlikely that these issues would have been discussed.

### Community structure capacity to engage with the local health system

As described in the Methods section, we assessed the capacity of community-level committees to engage with the local health system through the use of the Health Institution Capacity Assessment Process (HICAP). Using the HICAP, data were collected during baseline and post-baseline periods on six aspects of the committees’ ability to fulfil their roles: (1) Support to CHW Peer Supervisors and CHWs; (2) Use of health information in planning; (3) Regular and systematic supervision of CHW Peer Supervisors; (4) Monthly and quarterly meetings in which supervision is an agenda item; (5) The process of reviewing and contributing to CHW activity plans; (6) Community perception of the committee. Results of the HICAP analysis for these six indicators showed that the difference in differences (DID) in HMC capacity between intervention and comparison areas were not statistically significant, as shown in **Table 4**. **Table 5** indicates that the same analysis conducted on WDC scores showed a statistically significant difference for only one item: “Monthly and quarterly meetings in which CHW/Peer Supervisor activities is an agenda item” increased from 31.3% to 47.5% in the intervention area and decreased from 37.3% to 30.7% in the comparison area (*P* = 0.035 for the DID).

**Table 4:** HICAP scores for Ward Development Committees at baseline and endline for the intervention and comparison areas

Indicator	Intervention category	Baseline (%)			Endline (%)	Difference (in percentage points)*	Statistical significance	Greatest improvement (or least decline)
WDC support to CHW Peer Supervisors and CHWs	Intervention area	40.0% (32/80)			58.8% (47/80)	+18.8%	*P* = 0.027	Intervention area
	Comparison area	41.3% (31/75)			45.3% (34/75)	+4.2%	*P* = 0.742	
	Difference in differences†					+14.8%	ns	
WDCs use of health information in planning	Intervention area	48.8% (39/80)			61.3% (49/80)	+12.5%	*P* = 0.152	Intervention area
	Comparison area	49.3% (37/75)			53.3% (40/75)	+4.0%	*P* = 0.744	
	Difference in differences					+8.5%	ns	
Regular and systematic supervision of CHW Peer Supervisors	Intervention area	32.5% (26/80)			50.0% (40/80)	+17.5%	*P* = 0.036	Intervention area
	Comparison area	34.7% (26/75)			34.7% (26/75)	0.0%	*P* = 1.000	
	Difference in differences					+17.5%	ns	
Monthly and quarterly meetings in which supervision is an agenda item	Intervention area	31.3% (25/80)			47.5% (38/80)	+16.2%	*P* = 0.052	Intervention area
	Comparison area	37.3% (28/75)			30.7% (23/75)	-6.6%	*P* = 0.491	
	Difference in differences					+22.8%	*P* = 0.035	
WDCs review and contribute to CHW activity plans	Intervention area		46.3% (37/80)	52.5% (42/80)		+6.2%	*P* = 0.527	Intervention area
	Comparison area		40.0% (30/75)	37.3% (28/75)		-2.7%	*P* = 0.867	
	Difference in differences					+8.9%	ns	
Community perception of WDC	Intervention area		83.8% (67/80)	85.0% (68/80)		+1.2%	*P* = 1.000	Intervention area
	Comparison area		69.3% (52/75)	58.7% (44/75)		-10.6%	*P* = 0.234	
	Difference in differences					+11.8%	ns	

WDC – Ward Development Committee, HICAP – Health Institution Capacity Assessment Process, CHW – community health worker

*Endline minus baseline.

†Invervention area minus comparison area.

**Table 5:** HICAP scores for Health Management Committees at baseline and endline for the intervention and comparison areas

Indicator	Intervention category		Baseline (%)		Endline (%)	Difference (in percentage points)*	Statistical significance	Greatest improvement (or least decline)
HMC support to CHW Peer Supervisors and CHWs	Intervention area		65.3% (49/75)		61.3% (46/75)	-4.0%	*P* = 0.735	Comparison area
	Comparison area		49.3% (37/75)		53.3% (40/75)	+4.0%	*P* = 0.744	
	Difference in differences†					-8.0%	ns	
HMCs use of health information in planning	Intervention area		60.0% (45/75)		53.3% (40/75)	-6.7%	*P* = 0.510	Comparison area
	Comparison area		46.7% (35/75)		50.7% (38/75)	+4.0%	*P* = 0.744	
	Difference in differences					-11.7%	ns	
Regular and systematic supervision of CHW Peer Supervisors	Intervention area		34.7% (26/75)		33.3% (25/75)	-1.4%	*P* = 1.000	Comparison area
	Comparison area		33.3% (25/75)		46.7% (35/75)	+13.4%	*P* = 0.133	
	Difference in differences					-14.8%	ns	
Monthly and quarterly meetings in which supervision is an agenda item	Intervention area		34.7% (26/75)		48.0% (36/75)	+13.3%	*P* = 0.135	Comparison area
	Comparison area		30.7% (23/75)		50.7% (38/75)	+20.0%	*P* = 0.020	
	Difference in differences					-6.7%	ns	
HMCs review and contribute to CHW activity plans	Intervention area	34.7% (26/75)		41.3% (31/75)		+6.6%	*P* = 0.501	Comparison area
	Comparison area	32.0% (24/75)		46.7% (35/75)		+12.0%	*P* = 0.094	
	Difference in differences					-5.4%	ns	
Community perception of HMC	Intervention area	74.7% (56/75)		68.0% (51/75)		-6.7%	*P* = 0.470	Comparison area
	Comparison area	73.3% (55/75)		68.0% (51/75)		-5.3%	*P* = 0.591	
	Difference in differences					-1.3%	Ns	

HMC – Health Management Committee, HICAP – Health Institution Capacity Assessment Process, CHW – Community Health Workers

*Endline minus baseline.

†Intervention area minus comparison area.

### CHW reporting rates

Over the 34 months of the OR Study period CHWs submitted a total of 14 838-monthly reports and Peer Supervisors submitted 2409 reports (from both the intervention and comparison areas).We think it is reasonable to consider the submission of a monthly report as a proxy for CHW activity level, but we do not have any information on how active CHWs were that did not submit reports. As shown in **Table 6** and Appendix S4, Table S2 in **Online Supplementary Document**, during the post-baseline period CHWs were significantly more active in the intervention area than in the comparison area. Monthly CHW reporting data shows that a greater percentage of intervention area CHWs and Peer Supervisors were active as compared those in the comparison area. Compared to baseline levels, there was a net increase of 13.5 percentage points (*P* = 0.003) in reporting rates for CHWs and an increase of 8.5 percentage points for Peer Supervisors (not statistically significant). In addition, there was a net gain of 14.2 percentage points (*P* = 0.000) in the percentage of targeted households visited in the intervention area relative to the comparison area. These three indicators, which served as proxies for CHW functionality, were the only indicators which were discussed with frequency in the CHDRs, as shown in **Table 6****.**

**Table 6:** Effect of the Community Health Development Review Meetings on CHW reporting rates

Parameter	Results		
**Difference in differences (expressed in percentage points)**	**Area in which performance is more favorable**	**Presence of statistical significance**
Functionality of CHWs (based on changes between baseline and post-baseline in reports submitted):			
Frequency with which CHWs submit monthly reports to their peer supervisor	13.5	Intervention	*P* = 0.003
Frequency with which Peer Supervisors submit reports	8.5	Intervention	*P* = 0.498
Percentage of homes visited by CHWs (as measured from submitted reports)	14.2	Intervention	*P* < 0.001
Summary	All 3 differences are more favorable for the intervention area, and 2 out of these 3 are statistically significant.		

CHW – community health worker

### CHW home visitation quality as perceived by household members

As part of the endline household survey, respondents in both the intervention and comparison areas were asked about their experiences regarding CHW home visits. As shown in Table 7 and Appendix 4. Table 2, these measures of CHW functionality were more favorable in the intervention area for 8 out of the 11 measures. The difference between the intervention and comparison area for 2 of these indicators was statistically significant, and that for a third measure approached statistical significance. None of the 3 differences that favored the comparison area were statistically significant. Baseline levels of these indicators were not measured, so DID analyses were not possible.

**Table 7:** Summary of quality and effect of community health worker home visits

Parameter	Results		
**Difference in differences (expressed in percentage points)**	**Area in which performance is more favorable**	**Presence of statistical significance**
**Functionality of CHWs (based on differences in CHW activities between the intervention and comparison areas at endline):**			
Awareness of CHWs in the community	3.6	Intervention	No (*P* = 0.125)
Has ever had a CHW visit	2.2	Intervention	No (*P* = 0.954)
Has had CHW visit during pregnancy or in first 6 months of life of newborn	1.2	Intervention	No (*P* = 0.954)
Has had CHW visit at least once a month	0.9	Comparison	No (*P* = 0.830)
CHWs visits usually monthly	2.1	Intervention	No (*P* = 0.479)
CHW visits at least 20 min	1.9	Intervention	No (*P* = 0.476)
All functions performed by CHW during most recent visit	0.3	Comparison	No (*P* = 1.000)
CHW referred mother or child	6.3	Intervention	Approaching (*P* = 0.076)
Rating of CHW performance by mother	3.6	Comparison	No (*P* = 0.246)
Sought care from facility for child illness	13.3	Intervention	Yes (*P* < 0.001)
Sought care from multiple sources for illness	18.5	Intervention	Yes (*P* < 0.001)
Section summary	8 of 11 differences are more favorable in the intervention area. 2 of the 11 are statistically significant (*P* < 0.001), and 1 approaches statistical significance (*P* = 0.076). None of the differences that favor the comparison area are statistically significant.		
**Improved household behaviors:**			
Birth preparedness	0.8	Intervention	No (*P* = 0.873)
Immediate breastfeeding of newborn	2.8	Intervention	No (*P* = 0.595)
Feeding colostrum	5.7	Intervention	Yes (*P* = 0.043)
Exclusive breastfeeding	0	N/A	N/A
Continued breastfeeding	6.8	Comparison	No (*P* = 1.740)
Infant and young child feeding	18.3	Intervention	Yes (*P* = 0.002)
ORT use	6.4	Intervention	No (*P* = 0.310)
Summary	5 out of these 7 differences are more favorable for the intervention area, and 2 out of these 5 are statistically significant. The one difference favoring the comparison area is not statistically significant.		
**Improved care-seeking behaviors:**			
Contraceptive use	10.4	Intervention	Yes (*P* = 0.047)
Facility birth	6.9	Intervention	No (*P* = 0.132)
Care seeking for diarrhea	9.5	Intervention	Approaching (*P* = 0.079)
Treatment of diarrhea with ORT and zinc	4.9	Intervention	No (*P* = 0.365)
Care seeking for pneumonia	4.8	Comparison	No (*P* = 1.544)
Care seeking for malaria	0.4	Comparison	No (*P* = 1.054)
Section summary	4 out of these 6 differences are more favorable in the intervention area, 1 out of these 4 measures is statistically significant, and 1 approaches statistical significance (*P* = 0.079). Neither of the two differences favoring the comparison area are statistically significant		
Grand summary	17 out of 24 differences favor the intervention area, 5 out of 20 are statistically significant (*P* < 0.050), and 2 approach statistical significance (*P* > 0.50 and <0.010). None of the 5 differences that are *less* favorable in the intervention area are statistically significant.		

### Changes in household health-related practices

As described in the methods section, a knowledge, practice, and coverage (KPC) survey was conducted at baseline (May 2015) and endline (April 2017). A DID analysis was carried out for household behavior indicators that could have been influenced by CHWs. The DID was more favorable in the intervention for 5 out of 7 household behavior indicators, and 2 out of the 5 were statistically significant. Neither of the differences favoring the comparison area were statistically significant (**Table 5** and Appendix S4, Table S3 in **Online Supplementary Document**).

### Changes in care-seeking behaviors

Six indicators of care-seeking behaviors promoted by CHWs were also measured in the KPC surveys described above. As Table S4 of Appendix S4 in **Online Supplementary Document** indicate, 4 out of these 6 DID measures are more favorable in the intervention area, 1 is statistically significant, and 1 approaches statistical significance. The 2 indicators for which the DID measures favoured the comparison area were not statistically significant.

For all 4 categories of measures of community-based health system functioning described above, 20 of the 27 differences favour the intervention area, 7 out of the 20 are statistically significant, and 2 approach statistical significance. None of the differences favouring the comparison area are statistically significant or approached statistical significance.

## DISCUSSION

This paper has presented the results of a cluster-randomized controlled trial to determine the effect of a participatory community-based health information system (PCBHIS) using volunteer CHW-collected morbidity and mortality data and verbal autopsy results.

Implemented under challenging conditions of cholera and Ebola epidemics, the study provides evidence of moderate effectiveness of the PCBHIS intervention in:

- improving CHW functionality,
- improving healthy household behaviors and healthcare-seeking behaviors, as well as
- strengthening the capacity of Ward Development Committees to fulfil their roles.

If the same intervention could be implemented in a more stable setting, we think it is highly likely that the PCBHIS intervention could be even more effective. Thus, we consider this OR project for assessing the effectiveness of engaging communities to strengthen CHW performance to be a pilot project that hopefully others will want to adapt for replication under more favorable circumstances.

Results show that CHW functioning, which was the primary point of discussion in CHDRs, improved significantly. Other improvements were more modest. However, the weight of the evidence clearly favors a positive effect of the PCBHIS intervention. Given the implementation and contextual challenges encountered and the relatively brief period of intervention implementation (20 months), we view these findings as encouraging and therefore recommend that the approach to community engagement described here be replicated and further measured under more favorable circumstances.

Our findings indicate that meaningful community engagement can be achieved in a low-income, urban setting with favorable results for CHW functioning, for community capacity, and for key household-level MNCH practices. The results suggest that communities can contribute to the effectiveness of community-based health services. With proper facilitation, the approach described here has the potential to improve community capacity for processing local health data and mobilizing community action that will improve health services and health outcomes. In a companion article, we report the process of community engagement in conducting and reviewing verbal autopsies of child deaths [14].

It is unclear why the intervention area HMCs did not show evidence of improved capacity like the WDCs. One contributing factor may be that WDCs have not traditionally been engaged on health issues in the community, and this new role enabled them to develop their capacity. Another possibility is that the cholera and Ebola epidemics, some of which took place in the baseline period, built the capacity of the HMCs so that the subsequent HICAP/CHDR meetings did not have a measurable effect on HMC functionality. More research is needed on the differing motivations and factors affecting the capacities and actions of WDCs and HMCs, particularly as Sierra Leone seeks to further promote community engagement to strengthen its health system.

### Limitations and challenges

This study experienced significant challenges and limitations. Delays in implementation caused by the delays in the government’s formulation of its national CHW policy and by cholera and Ebola epidemics caused serious operational challenges for the study. Most significantly, we were not able to address original research questions on the effect of the PCBHIS on under-five mortality. The low rate of report submission from CHWs along with underreporting of vital events even for those who submitted reports made it impossible to accurately monitor changes in mortality in the intervention area, as had been planned originally. The abbreviated implementation period and the presence of a well-run child survival project in the comparison area both made it unlikely that a mortality impact in the intervention area would have occurred even if mortality rates has been measured accurately.

We had also hoped to observe stronger improvements in coverage of household-level maternal and child health behaviors and practices in the intervention area relative to the comparison area than we did, as behavior change communication was a key role of CHWs. However, the limited functionality of the CHW program, the shortened time period of intervention implementation, and the high rates of in- and out-migration in these urban communities all contributed to less than optimal health data for review at the CHDR meetings and for less than optimal capacity of the community to promote strengthened community-based services. In complex systems, such as community and social systems, effects of interventions may fit longer timelines than afforded by OR designs [15,16].

### Capacity building, community engagement and strengthening of community-based health services: other reported experiences

Existing evidence suggests that community engagement can support improved functioning of government health facilities [16], reduce incidence of key childhood illnesses [17] and improve the quality of health programming [18]. Yet the literature calls for more evidence of effectiveness of community engagement [19,20].

A PCBHIS built on community-based surveillance is one approach to strategically support and engage communities to collect, analyze and act on local health data. The PCBHIS developed for this study centered on household-level epidemiological, morbidity and/or mortality data gathered by CHWs. While using health data for decision making is not a new approach (USAID led a project called Data for Decision Making for the Health Sector beginning in 1991), these activities have usually focused on higher-level health officials using data collected by paid, trained research assistants directed by a vertical national program. The limited use of the data-for-decision making approach for community-based maternal and child survival programs that has been published in the peer-reviewed literature has not undergone the level of rigorous assessment that we present here. Few studies and interventions such as ours have focused on providing access to data gathered through a local CBHIS to the members of the community from which it was gathered. Community engagement can facilitate ownership of locally collected data [21], and it can have positive impacts on health and nutrition indicators [22]. However, we have not identified any evidence for this from slum communities in low-income countries.

Integral to the functionality of the CBHIS is the quality of the surveillance data. In our study, volunteer CHWs with other responsibilities provided this data. Various studies have found that CHWs can be used effectively to collect community-based surveillance data [23], and that this data provides an important complement to data coming from health facilities, which can be incomplete since it relies only on those coming to the facilities for care [24,25].

CHW programs often have challenges with quality and coverage, particularly in volunteer programs [26,27], and CHW performance has been specified as a key element of high quality CHW programming [28]. Several studies have found that community engagement and support can lead to better CHW performance and motivation [29,30]. However, documentation of approaches and effects of community engagement on CHW performance such as those we report here remain scarce.

Sierra Leone faces a unique opportunity, with the country now stabilized after the catastrophic Ebola outbreak, with the government and donors supportive of CHW programming and community engagement, with a stronger new national CHW program, and with newly finalized guidelines for Health Management Committees to help formalize these structures and standardize their operation. Establishing a functional PCBHIS linked to the work of CHWs and including a practical, low-cost intervention such as Community Health Data Review meetings may allow Sierra Leone, and other countries with similar contexts, to enable the community and health system to work more effectively together for improved health outcomes.

Nonetheless, valuable lessons can be extracted from the implementation of this intervention and its evaluation. While CHW visitation coverage during the post-baseline period was significantly higher in the intervention area (42.7% as compared to 24.2% in the comparison area), the lack of CHW visits in more than half of the households presents obvious limits for the overall effectiveness of the CHW program. However, this does not negate the positive influence of the PCBHIS intervention itself on the functionality of the CHW program.

For practitioners and policy makers seeking to address the difficult challenge of CHW motivation through non-financial means, the CHDR meetings present a practical solution, with the added benefit of meaningful community engagement. While it is yet to be seen if the PCBHIS in this context can be used to address and influence trends in serious morbidity and mortality, our findings demonstrate that communities are able to engage with data, determine actions, and motivate volunteer CHWs to become more active.

## CONCLUSIONS

The operations research project reported here provides encouraging evidence regarding the relevance of community engagement and community-based health programming for strengthening health systems in low-income urban contexts. The continued rapid growth of urban, slum populations will require increasing attention to approaches to improving the health systems in these settings. Implementing a participatory community-based health information system could become one such practical and affordable approach.

## Additional material

Online Supplementary Document
