# Supplementary material for: Piloting a participatory, community-based health information system for strengthening community-based health services: findings of a cluster-randomized controlled trial in the slums of Freetown, Sierra Leone
Source: J Glob Health. 2019 Feb 21;9(1):010418. doi: 10.7189/jogh.09.010418 (PMC6394878; doi:10.7189/jogh.09.010418)
Supplement: Online Supplementary Document [file jogh-09-010418-s001.pdf]

O'Conner et al. J Glob Health 2019; 1: 010418

Appendix S1.
Supplemental table

Table S1. Child survival project objectives and main activities

Project objective	Activities
Improve the quality of MNCH delivered at health facilities.	Training government health facility staff on key MNCH guidelines and treatment protocols<br>Coaching and mentoring by clinical staff members in ten government health facilities. Areas of focus included clinical service delivery, record keeping, and human resources<br>Provision of job aids and essential medical supplies.
Increase household preventive practices , awareness of danger signs and appropriate care seeking behavior for childhood illness and maternity-related problems	Development of research-based behavior change communication materials to be used by CHWs<br>Training 1,219 volunteer CHWs and 106 volunteer Peer Supervisors<br>Working with CHWs to make monthly home visits to a caseload of 25 households each and collect vital event and morbidity data
Strengthening community and district capacity to plan, manage, and monitor health activities.	Facilitating training of Ward Development Committees by Freetown City Council on roles and responsibilities<br>Facilitating training of Health Management Committees by District Health Management Team on roles and responsibilities<br>Implementing participatory Health Institution Capacity Assessments every 6-9 months with each HMC and WDC
Advocate for improved national-level MNCH policy and improved coordination at the local government level.	Strong engagement in the development of materials for implementation of the MOHS 2012 CHW policy<br>Strong engagement in the development of the 2017 National CHW Policy

Appendix S2.
Challenges in the implementation of the child survival project (CSP) and operations research (OR) study

Challenges were encountered related to the implementation of the CSP and the OR Study, limiting the ability of the OR Study to answer original research questions. Challenges were caused primarily by unexpected disease outbreaks in the study area, which limited CHWs, HMCs and WDCS to fully fulfil their roles, and delays in the MOHS in the finalization of its national CHW Policy, which reduced implementation timelines and effected CHW motivation. These events and processes effected the CSP and OR Study in multiple ways, but most significantly created delays in implementation of activities and led to CHWs reporting at lower than anticipated quality and completeness. While the project received a no-cost extension to support implementation for a further six months, implementation of the OR Study remained shorter than originally anticipated. Appendix 2. Table 1 below outlines the interventions the OR Study had originally planned and what actually took place.

Appendix S2. Table S1. Planned versus actual intervention activities of the operations research study

Activity	Planned	Actual (with reasons for changes)
CHW intervention: duration of OR study period	Data collection based on CHW household visits would start in early year 2, taking place over a 42-month period. .	CHW intervention ran for between 21 to 34 months (start date varied by community). Delays were related to the CSP and its CHW activities needing to align with the national CHW policy. Therefore CHW recruitment and training could not take place until the policy and training and job aid materials were finalized in early Year 3 of the CSP. Following this, the Ebola outbreak caused the project to suspend rolling out CHWs into additional communities until mid-Year 3. CHWs in all 10 communities were trained and household visits began in 4 of the 10 communities prior to this suspension
CHW intervention: reporting rates	We anticipated that CHWs would be active and would provide monthly reports for analysis.	Average reporting rates for the duration of the CHW intervention were approximately 40%, severely limiting the extent to which the PCBHIS could indicate morbidity and mortality trends. Challenges to motivation of CHWs were multiple: lack of financial incentives, non-financial incentives such as ID cards and certificates of training from MOHS being promised but not ultimately provided, fear amongst CHWs, CSP and OR Study staff to make household visits during the initial stages of the Ebola outbreak, and frequent engagement of CHWs in Ebola response activities (which paid well), or demotivation due to the fact that some CHWs were not selected to implement such activities, both of which took the focus away from the routine ongoing CHW role.

Appendix S2. Table S1. Planned versus actual intervention activities of the operations research study		
**Activity**	**Planned**	**Actual (with reasons for changes)**
Timing and frequency of Community Health Data Review (CDHR) meetings	To start quarterly CHDR meetings early in Year 3 once PCBHIS tools were finalized and following a 3-month pilot period, allowing for approximately 30 months of implementation.	The CHDR meetings did not begin until mid-Year 4 of the CSP and took place over a period of 20 months, rather than the anticipated period of 30 months. Instead of designing and piloting its own PCBHIS tools (as was the initial plan), the OR Study was required to use MOHS CHW tools, including the CHW monthly report form. CHW monthly report forms were not finalized by the MOHS until early in Year 3. Since the beginning of CHW home visits in all communities was delayed and there were lower than expected CHW reporting rates, the initiation of the CHDRs had to be delayed since they were initially planned to review CHW-gathered health data. Once the CHDR meetings began, we increased the frequency from quarterly to bimonthly in an attempt to enhance the impact of the OR Study intervention in a shorter time period.
Content of CHDRs: CHW data	Community structures would review CHW-gathered health data to determine the most urgent health issues in their community and develop actions to address these.	CHW-gathered health data were reviewed in CHDR meetings, but meeting content mostly focused on rates of CHW and Peer Supervisor reporting, number of households reached, and how to increase these, with some discussion attempted on data quality. Discussions on health-related findings from the CHW data were limited. Changes in CHW-data content were due to persistent low levels of quality and completeness of CHW-gathered data. Reasons for low reporting completeness are discussed above. Low data quality stemmed from persistent challenges by CHWs to use the monthly reporting forms due to a lack of a standardized user guide for the forms from MOHS, lack of instructions appropriate for CHWs and Peer Supervisors with low literacy levels, delays between initial CHW training and initiation of household visits in some communities due to onset of Ebola outbreak, and the CSP and OR study prioritizing issues around low reporting rates rather than quality of reporting. There were some reports of resistance by CHWs to report deaths and some illnesses, particularly diarrhea, due to fear around association with Ebola, even following the end of the outbreak.
Content of CHDRs: verbal autopsy results	Community structures would review verbal autopsy results to determine the most frequent causes of death of under-5 children in their community and develop actions to address these.	Discussions of specific cause of death as determined by verbal autopsies were limited. Themes of verbal autopsy narratives and case studies of verbal autopsies were discussed and were the subject of great interest. Actions were developed to address findings. The OR Study team observed that CHDR participants were more able to recommend actions in response to the qualitative narrative themes arising from the verbal autopsy rather than in response to actual cause of death data.

Changes in the plans for the implementation of the CSP and OR Study also led to changes in monitoring and evaluation (M&E) activities. Some originally planned M&E activities were not possible or appropriate. In other cases, monitoring data not previously planned to be used emerged as more appropriate for assessing results of the PCBHIS. Table 2 below summarizes these changes.

Appendix S2. Table S2. Planned versus actual monitoring and evaluation activities

Activity	Planned	Actual (with reasons for changes)
Health Institution Capacity Assessment Process (HICAP) scores	To be used for CSP monitoring, not originally designed to be used to determine OR Study results.	HICAP scores used to evaluate effect of PCBHIS on community structure capacity to engage with the local health system and fulfill other functions. The data set from the originally intended tool for assessing community capacity (the PRISM Organizational and Behavioral Assessment Tool) was not used as baseline was conducted two years before the OR intervention began.
Knowledge, practice and coverage surveys	Designed to evaluate changes in health knowledge, practices, and coverage of facility-based heath interventions in CSP implementation area.	Questions on coverage and quality of CHW interventions added to final KPC survey to determine differences between intervention and comparison areas.
Monthly CHW reporting data	Internal monitoring only.	Used to evaluate the effect of the PCBHIS since the low rates of CHW reporting turned out to be a major issue.

Appendix S3.
Photographs of the operations research study intervention

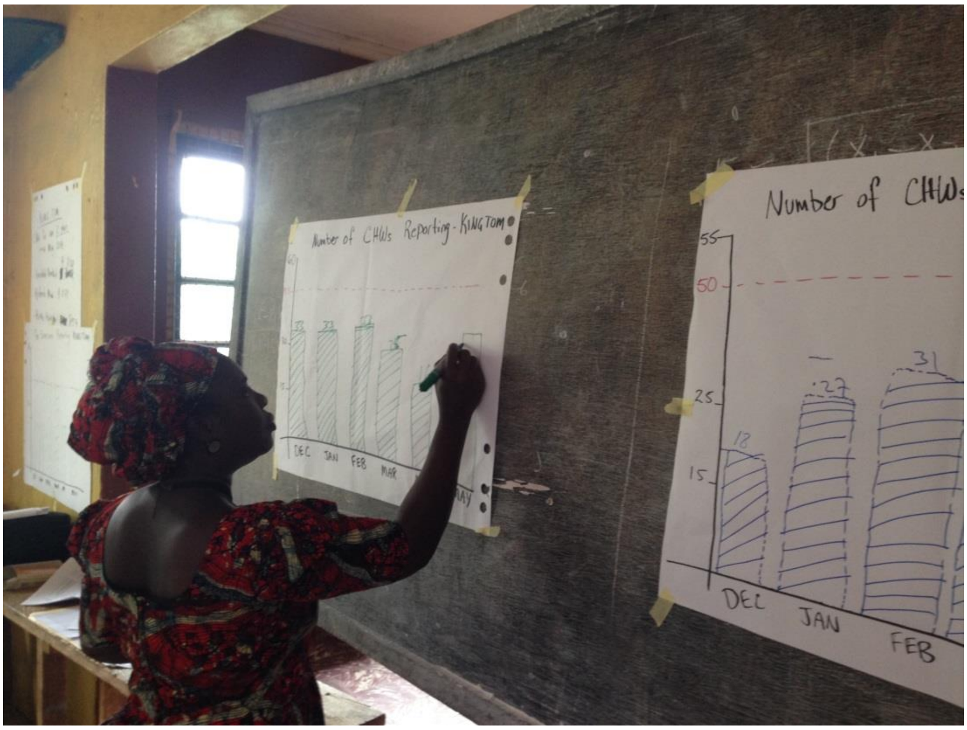

Appendix 3. Figure S1. Kingtom community Peer Supervisor Rugiatu Mansaray drawing bar graph of the number of CHWs reporting at a CHDR, June 2016. A graph showing neighboring community Grey Bush CHW reporting can be seen as well.

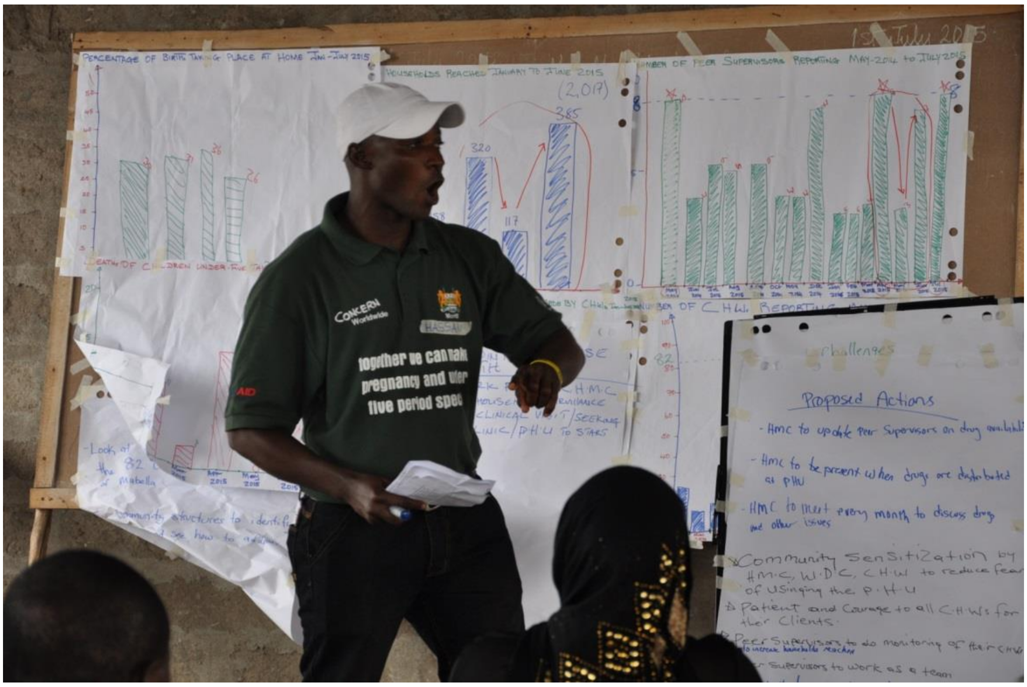

Appendix 3. Figure S2: Mabella Health Management Committee Member Hassan Sesay facilitating the discussion of action points at a Community Health Data Review meeting, August 2015

Appendix S4. Supplemental tables

Appendix 4. Table S1. Measures of functionality of the CHW program

Parameter	Intervention area	Comparison area	Difference (intervention area minus comparison area)	Statistical significance of difference	Greatest improvement (or least decline)
**Awareness of CHWs in Community** (Percentage of mothers of children who are aware of CHW in the community)	76.0% (288/379)	72.4% (299/413)	3.6%	p=0.125	Intervention area
**Has ever had a home visit from a CHW** (Percentage of mothers of children who have ever had a visit from a CHW)	67.8% (257/379)	65.6% (271/413)	2.2%	p=0.954	Intervention area
**Has had a visit from a CHW in the time of their pregnancy and/or life of their youngest child age 0-<6 months of age** (Percentage of mothers of children who have had a visit from a CHW in the last year)	58.2% (64/110)	57.0% (61/107)	1.2%	p=0.891	Intervention area
**Has a visit from a CHW at least once a month** (Percentage of mothers of children who has a CHW visit on at least a monthly basis)	44.1% (167/379)	45.0% (186/413)	-0.9%	p=0.830	Comparison area
**Continuity in the CHW who visits** (Percentage of mothers of children who generally have a HH visit on a monthly basis from the same CHW)	19.0% (72/379)	16.9% (70/413)	2.1%	p=0.479	Intervention area
**Adequate duration of CHW visit** (Percentage of mothers of children 0-5months who have a HH visit from the same CHW on a monthly basis which is at least 20 minutes)	6.3% (24/379)	4.4% (18/413)	1.9%	p=0.267	Intervention area
**CHW performance during HH visit** (Percentage of mothers of children who had a HH visit from a CHW in the last year in which the CHW performed all roles)	20.8% (79/379)	21.1% (87/413)	-0.3%	p=1.000	Comparison area
**CHW referral rate** (Percentage of mothers of children h had a HH visit from a CHW in the last year which resulted in the CHW referring the mother or child to the health facility)	53.8% (204/379)	47.5% (196/413)	6.3%	p=0.076	Intervention area
**Perception of performance of CHW by mother of child 0-<6 months of age** (Percentage of mothers of children who have had a visit from a CHW in the past year who found the visit helpful or somewhat helpful)	67.8% (257/379)	71.7% (296/413)	-3.9%	p=0.246	Comparison area
**Appropriate initial source of treatment/advice for all illnesses of children 0-59 months** (Percentage of children age 0-23months with any illnesses in last two weeks who sought initial care from a health facility, including a government facility, private facility, or hospital)	57.1% (194/340)	43.8% (166/379)	13.3%	p=0.000	Intervention area
**Care seeking from multiple sources** Percentage of mothers of children age 0-23 months with an illness in last two weeks who sought care or advice from multiple sources	77.9% (265/340)	59.4% (225/379)	18.5%	p=0.000	Intervention area

Appendix 4. Table S2. CHW and Peer Supervisor reporting rates, baseline to post-baseline, intervention versus comparison areas

Parameter	Intervention category	Baseline period (%)	Post-baseline period (%)	Difference (in percentage points)	Statistical significance	Greatest improvement (or least decline)
**CHW reporting rate***<br>(Number of CHWs reporting / number of CHWs trained)	Intervention area	35.6% (138/388)	46.6% (237/509)	+11.0%	p<0.001	Intervention area
	Comparison area	40.6% (161/397)	38.1% (271/710)	-2.5%	p=0.441	
	Difference in differences			+13.5%	p=0.003	
**Peer Supervisor reporting rate***<br>(Number of Peer Supervisors reporting / number of Peer Supervisors trained)	Intervention area	74.1% (26/35)	79.6% (39/49)	+5.6%	p=0.605	Intervention area
	Comparison area	76.9% (24/31)	74.0% (42/57)	-2.9%	p=0.800	
	Difference in differences			+8.5%	p=0.498	
**Percentage of community covered by CHW home visit**<br>(Households visited / (households in the community * number of reporting months))	Intervention area	17.4% (21,722/ 124,751)	42.7% (94136/ 220,689)	+25.3	p=0.000	Intervention area
	Comparison area	13.1% (18,606/ 141,724)	24.2% (91,764/ 379,953)	+11.1%	p=0.000	
	Difference in differences			+14.2+14.2%	p=0.000	

Appendix 4. Table S3. Results of key household level survey results on MNCH practice, change from baseline to post-baseline, intervention area versus comparison area

Indicator	Intervention category	Baseline (%)	Endline (%)	Difference (in percentage points)	Statistical significance	Greatest improvement (or least decline)
**Birth preparedness**: *Percentage of mothers of children 0-23months who made preparations before the birth of their youngest child*	Intervention area	42.5% (127/299)	62.8% (238/379)	+20.3%	p=0.000	Intervention area
	Comparison area	35% (105/300)	54.5% (225/413)	+19.5%	p=0.000	
	Difference in differences			+.8%	p=0.873	
**Immediate breastfeeding of newborns**: *Percentage of children age 0-23 months who were put to the breast within one hour of delivery*	Intervention area	56.2% (168/299)	74.9% (284/379)	+18.7	p=0.000	Intervention area
	Comparison area	48.7% (146/300)	64.6% (267/413)	+15.9	p=0.000	
	Difference in differences			+2.8	p=0.595	
**Feeding colostrum**: *Percentage of children age 0-23 months who were fed colostrum after birth*	Intervention area	87% (260/299)	96% (364/379)	+9.0%	p=0.000	Intervention area
	Comparison area	92.3% (277/300)	95.6% (395/413)	+3.3%	p=0.063	
	Difference in differences			+5.7%	p=0.043	
**Exclusive breastfeeding:** *Percentage of children 0-5 months who were exclusively breastfed during the last 24 hours*	Intervention area	32.2% (28/87)	30.0% (33/110)	-2.2%	p=0.740	No difference
	Comparison area	36.8% (32/87)	34.6% (37/107)	+2.2%	p=0.750	
	Difference in differences			0	p=0.998	
**Continued breastfeeding 6-23 months:** *Percent of children 6-23 months who are still breastfeeding*	Intervention area	65.5% (139/212)	64.7% (174/269)	-0.8%	p=0.855	Comparison area
	Comparison area	65.3% (139/213)	71.2% (218/306)	5.9%	p=0.154	
	Difference in differences			-6.8%	p=1.740	
**Infant and young child feeding**: *Percent of infants and young children 6-23months fed according to a minimum of appropriate feeding practices*	Intervention area	34.4% (73/212)	40.5% (109/269)	+6.1%	P=0.171	Intervention area
	Comparison area	35.7% (76/213)	23.5% (72/306)	-12.2%	p=0.003	
	Difference in differences			+18.3%	p=0.002	

Indicator	Intervention category	Baseline (%)	Endline (%)	Difference (in percentage points)	Statistical significance	Greatest improvement (or least decline)
**ORT use**: *Percentage of children 0-23 months with diarrhea in the last two weeks who received oral rehydration solution (ORS) and/or recommended home fluids*	Intervention area	65.1% (123/189)	73.8% (175/237)	+8.7%	p=0.052	Intervention area
	Comparison area	73.1% (117/160)	75.4% (175/232)	+2.3%	p=0.608	
	Difference in differences			+6.4%	p=0.310	

Appendix 4. Table S4. Results of key household level survey results on illness care seeking, change from baseline to post-baseline, intervention area versus comparison Area

Indicator	Intervention category	Baseline (%)	Endline (%)	Difference (in percentage points)	Statistical significance	Greatest improvement (or least decline)
**Current contraceptive use among mothers of young children**: *Percentage of mothers of children age 0-23 months who are using a modern contraceptive method*	Intervention area	29.8% (89/299)	48.0% (182/379)	+18.2%	p=0.000	Intervention area
	Comparison area	42.3% (127/300)	50.1% (207/413)	+7.8%	p=0.039	
	Difference in differences			+10.4%	p=0.047	
**Facility birth:** *Percentage of last-born children age 0-23 months who were born in a health facility*	Intervention area	78.9% (239/299)	84.7% (321/379)	+5.8%	p=0.050	Intervention area
	Comparison area	88% (264/300)	86.9% (359/413)	-1.1%	p=0.663	
	Difference in differences			+6.9%	p=0.132	
**Care seeking for diarrhea**: *Percentage of children 0-23months with diarrhea in the last two weeks whose mothers sought outside advice or treatment for the illness*	Intervention area	77.8% (147/189)	86.1% (204/237)	+8.3%	p=0.025	Intervention area
	Comparison area	83.1% (133/160)	81.9% (190/232)	-1.2%	p=0.025	
	Difference in differences			+9.5%	p=0.079	
**Treatment with ORS and zinc**: *Percent of children 0-23months with diarrhea in the last two weeks who were treated with both ORS/recommended home fluids and zinc*	Intervention area	10.1% (19/189)	34.2% (81/237)	+24.1%	p=0.000	Intervention area
	Comparison area	11.9% (19/160)	31.0% (72/232)	+19.1%	p=0.000	
	Difference in differences			+4.9%	p=0.365	
**Care seeking for pneumonia**: *Percentage of children 0-23 months with chest-related cough and fast and/or difficult breathing in the last two weeks who were taken to an appropriate health provider*	Intervention area	58.1% (137/236)	80.3% (171/213)	+22.2%	p=0.000	Comparison area
	Comparison area	56.1% (101/180)	83.0% (142/171)	+26.9%	p=0.000	
	Difference in differences			-4.8%	p=1.544	

Indicator	Intervention category	Baseline (%)	Endline (%)	Difference (in percentage points)	Statistical significance	Greatest improvement (or least decline)
**Care seeking for malaria**: *Percentage of children aged 0-23 months with a febrile episode during the last two weeks who were taken to an appropriate place for treatment*	Intervention area	52.5% (146/278)	79.7% (208/261)	+27.2%	p=0.000	Comparison area
	Comparison area	51.4% (126/245)	79.0% (203/257)	+27.6%	p=0.000	
	Difference in differences			-0.4%	p=1.054	

Appendix S5. The Health Institution Capacity Assessment Process (HICAP) Instrument

Health Institution Capacity Assessment Process (HICAP)

HICAP Matrix

Developed by Concern Worldwide Sierra Leone in collaboration with members of Sierra Leone's Western Area Urban Health Management and Ward Development Committees, for the Western Area health sector context

What is HICAP?

The HICAP targets existing committees and organizations at the community and district level to assess, measure, and monitor local organizational capacity and to strengthen capacity through specific actions. It is a flexible, interactive tool and process that is used to create and achieve a vision of an ideal setting or system within a community. The HICAP is used to assess the present capacity of a committee through a baseline evaluation, to set capacity goals to achieve the vision, and to measure changes in capacity of the local committees.

The specific objectives of the HICAP are as follows:

- To create a shared understanding of the capacities required for the committee to fulfill its purpose to become a lasting institution within the community and to improve service delivery;
- To determine the committee's present position and target capacity position using the HICAP assessment scores in terms of overall capacity to provide [health] services to the citizens of the community;
- To create a list of actions detailing the steps to be taken for a committee to reach its target scores and, incorporate these into the current annual plan and future annual plans; and
- To establish a schedule to conduct follow-up assessments and track progress.

Capacity Areas to Assess:

Capacity Area I: Participatory Planning

The systems in place to ensure HMC/WDC activities are planned in advance, with proper division of responsibilities, phases of implementation, and input from all HMC/WDC members.

Capacity Area II: Leadership (Governance)

The processes followed to ensure the HMC/WDO remains representative of and responsible to the community, through proper internal management ensuring all members understand their responsibilities, good character, and fully participate in decision making to achieve a common goal.

Capacity Area III: Resource Mobilization and Management

The HMC/WDC ability to raise funds, locate and utilize resources and maintain proper financial records available to the public.

Capacity Area IV: Collaboration and Coordination

The WDC/HMCs ability to establish relationships with key community, district, and relation institutions, resulting in an increase of services in support of the community.

Capacity Area V: Monitoring and Evaluation

The WDC/HMC's ability to systematically document the results of its activities and ensure this information is regularly reviewed and used as the basis for future planning. The WDC/HMC actively supports the collection of community health data and uses relevant information to inform its planning process.

Capacity Area VI: Supervision

The WDC/HMC routinely and systematically supervise CHW Peer Supervisors and provide timely feedback on their performance. The WHC/HMCs are involved in and oversee the CHW training and activity plans and ensure these activities are in accordance with the HMC/WDC annual plans and respond to the needs of the community. The WDC/HMC are the liaison between the CHW Supervisors and PHU Staff.

Why Use HICAP?

This matrix can be used in combination with the Community Self-Assessment Score Card Booklet to measure, report, and monitor changes in capacity levels for each capacity area. At the first assessment, the matrix is used to assign a score based on the current or existing

capacity for each capacity area; this serves as the baseline score. After a baseline score is agreed upon, the committee will then create a target score for each capacity area, based on their vision. These scores should be documented at the beginning of the Community Self-Assessment Score Card Booklet.

Every six months, the committees are encouraged to revisit the HICAP matrix and reassess their capacity at that point in time and agree to a capacity score for each capacity area. By routinely collaborating to evaluate and asses each capacity area on a semi-annual basis this matrix can be used to compare current, baseline, previous, and target capacity scores for a given community, allowing a community to track and self-monitor their change in capacity over time for in each capacity area.

Capacity Area I: Participatory Planning

Definition: The systems in place to ensure HMC/WDC activities are planned in advance, with proper division of responsibilities, phases of implementation, and input from all HMC/WDC members.

Indicator	1st Stage 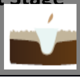	2nd Stage 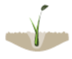	3rd Stage 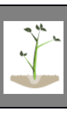	4th Stage 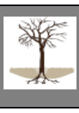	Fina
**1.1 Meeting Attendance**<br>(% of committee members present at every meeting	Very Poor<br>(3 people or less present)<br>WDC =11<br>HMC =15	Poor<br>(4-5 people present)	Moderate<br>(6-8 people present)	Good<br>(9-10 people present)	Exce<br>(11 (
**1.2 Regular Meetings with an Agenda and an elected chair to lead the meeting**<br><br>*Are there meetings held on a regular basis with a prepared agenda? Is a councilor present during the meetings?*	HMC/WDC meetings are not held	Meetings are held ad hoc and are often planned last minute. There is no prepared agenda. Minutes are not kept during the meeting and action points are not assigned to individuals nor due dates set. A councilor is not present during meetings	Meetings are held a few times a year but the day and time are not fixed and not much advanced notice is given. An agenda is prepared before the meeting but is not based on last meeting's action points. Some minutes are kept during the meetings but are not complete. Some action points are assigned to individuals with due dates set. A councilor is present during some meetings	There is a fixed day and time for monthly meetings. But changes occur often and giving proper advanced notice is not a priority. An agenda is prepared before the meeting based on prior meeting's action points. Minutes are kept during the meetings. Most action points are assigned to individuals with due dates set. The councilor is present most of the time	Mee<br>fixed<br>give<br>are (<br>give<br>mee<br>mee<br>prior<br>the r<br>actio<br>assig<br>set.<br>duri
**1.3 Written Quarterly or Annual Plan**<br><br>*Is there a written quarterly/annual workplan based on community priorities?*	There is no written quarterly or annual workplan	A simple quarterly or annual plan (i.e. no activity leaders assigned) is written with 1-2 committee members having input	A quarterly or annual plan is written with set targets and committee members assigned as activity leader. 3-5 committee members participate in planning discussions	A quarterly or annual plan is written with set targets and activity leaders are assigned. Some consideration for plans beyond current year. Annual Plan demonstrates a commitment to fundraising or asking for support for specific activities in the community. Most (6-8) committee members contribute to shared discussions and decision making for quarterly/annual plan	An a<br>targ<br>simp<br>curr<br>men<br>com<br>prep<br>Ther<br>activ<br>beer<br>men<br>deci

Capacity Area II: Leadership (Governance)

Definition: The processes followed to ensure the HMC/WDO remains representative of and responsible to the community, through proper internal management ensuring all members understand their responsibilities, good character, and fully participate in decision making to achieve a common goal.

Indicator	1st Stage 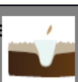	2nd Stage 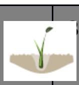	[illegible]rd Stage 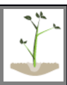	4th Stage 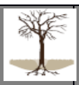	Final Stage 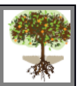
**2.1 Membership Election and Replacement Process**<br><br>*Is the process of replacing members fair and transparent?*	There is no constitution or guidelines which explain the membership term. Election/selection process is not participatory. Some members or outside entities have special influence. There is no replacement system for members who drop out or can't perform responsibilities. There is no consideration for ensuring new members meet specified constitution or guidelines for membership	Thought has been given to developing a constitution or guidelines to explain membership. Selection process is ad hoc and not transparent. Nominations and votes are often unfairly influenced by a few individuals. A system for replacement has been proposed. There is little consideration for ensuring new members meet criteria	A draft constitution or guidelines are developed. There are about 10 members of a committee plus the Paramount Chief of the Chiefdom and the councilor elected from that ward. Community members vote on new members but sometimes nominations and/or votes are unfairly influenced. There is a system in place to which is usually used to replace members who drop-out. There is some effort to ensure new members meet criteria	A transparent selection process has been defined with specific rules according to the constitution or guidelines which have been finalized. Community members elect members in a public meeting. There is little to no influence from other individuals. The committee sometimes replaces members who drop out and they communicate this decision to FCC/DHMT. Ensuring new members meet most of the criteria is a priority	The constitution or guidelines is adhered to at all times. There is a well-defined, transparent member replacement process in place. First priority is to ensure those nominated meet all criteria and are the most qualified from their representative group. The committee always replaces members in a timely manner who drop out and they communicate this decision to FCC/DHMT
**2.2 Committee roles assigned and understood?**<br>*Are committee roles (chairperson, vice-chairperson, secretary, treasurer), well defined, assigned and understood by those selected for the positions?*	No roles are defined or assigned	1-2 positions are assigned (i.e., chairperson and vice-chairperson). Roles are not well defined, the person does not have proper understandin g of it 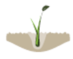	Some other positions are assigned. Most roles and responsibilities are well defined. Some individuals assigned a role do not have proper understanding of their responsibilities	Most positions are assigned. All roles and responsibilities are well defined. Most individuals assigned a role have a good understanding of their responsibilities	All roles are assigned. All responsibilities are well defined and documented. All individuals assigned a role have a good understanding of their responsibilities
**2.3 Demonstrated Leadership capacity**<br>*Has focal point been appointed for the discussion of community problems and*	No one in the committee has been appointed as a focal point to spearhead discussion of community issues. There is no evidence that any	A focal point has been identified to lead on discussions about community issues however	A focal point has been identified, and discussions on community problems or specific needs are taking place. Evidence has been gathered on	A focal point actively ensures action points have been developed to address the community problems and needs and	Measureable action has been taken. Preventative measures or steps have been incorporated into the quarterly/annual

Indicator	1st Stage 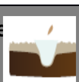	2nd Stage 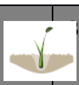	[illegible]rd Stage 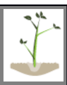	4th Stage 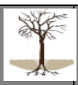	Final Stage 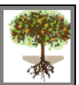
needs within the community. Remedial action is taken when necessary and recommendation s are made to the local council/DHMT?	recommendation s have been made to FCC or DHMT in communities where there are significant issues	these discussion are ad hoc and information is insufficient or lacking evidence	what the problems are and what are the exact needs in the community. Discussions on community problems are ongoing	stakeholders have been assigned to these points. Alternative recommendation s have been consider to resolve the problems and meet the needs in the community	work plan to review progress and ensure there is no reoccurrence. Recommendation s have been developed and shared with community stakeholders and incorporated into quarterly/annual plans
**2.4 Participatory Decision Making** *Do all committee members have equal opportunity to participate in decision-making?*	Chairperson makes decisions without consultation and without members' input/vote.	Chairperson sometimes consults with a few members for some decisions but always has final say.	Chairperson regularly consults with a few members to make decisions but the full committee rarely approached for input.	Discussions include most committee members, and Chairperson brings important decisions to the full committee for input/vote.	Discussions include all committee members and those directly impacted. Decisions include all committee members' vote and/or input from those directly impacted
**2.5 There is a 50/50 gender balance on the committee** *Is there equal representation by gender on the committee? Is there gender balance?*	There is no gender balance. The committee is all male or all female	Most of the members of the opposite gender are represented on the committee. Decisions are almost always made by one gender and the opposing gender feels silenced	An effort has been made to elect more of the underrepresente d gender and several new members have been recruited. 70%/30% gender balance. Decisions are sometimes made by one gender but the opposing gender has a voice	The committee is working to achieve a better gender balance and now the representation is 60%/40%. Occasionally one gender is favored over the other	Members of the committee 50% are women and 50% are male. Each member has an equal voice

Indicator	1st Stage 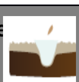	2nd Stage 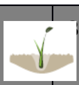	[illegible]rd Stage 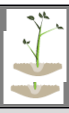	4th Stage 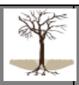	Final Stage 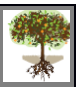
**2.6 Community Knowledge and Perception of WDC/HMC**<br><br>*Does the community know what the function of the HMC/WDC is and how does the community perceive the HMC/WDC?*	90% or more of the community are **unaware** of the HMC/WDC or consider it a shadow entity with members only interested in their image & status	At least 25% of the community members know the WDC/HMC and its purpose and services. Those who are aware of the WDC/HMC know its mission but do not feel members are truly dedicated to accomplish community development initiatives	At least 50% of community members know the WDC/HMC its purpose and services offered. Among those who are aware of the WDC/HMC, most feel the WDC/HMC has some dedicated members and is making some effort to implement valuable activities and changes	At least 75% of community members know the WDC/HMC, its purpose, and services. Those who know the HMC/WDC have mostly positive perceptions of the HMC/WDC and feel the committee members are dedicated to the HMC/WDC mission and they work to implement valuable activities/changes	Nearly everyone (at least 90%) in the community knows the HMC/WDC, its purpose and the services provided. Those who know the WDC/HMC feel WDC/HMC members are very dedicated to the people and feel their activities have impacted their lives. WDC/HMC has educated residents on their rights and obligations in relation to government policies (i.e., decentralization and free healthcare)

Capacity Area III: Resource Mobilization and Management

Definition: The HMC/WDC ability to raise funds, locate and utilize resources and maintain proper financial records available to the public

Capacity Area III: *Resource Mobilization and Management*

Indicator	1st Stage 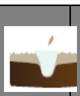	2nd Stage 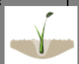	3rd Stage 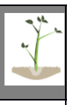	4th Stage 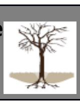	Final Stage 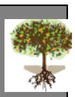
**3.1 Fundraising activities are implemented in the community and included in the Annual Plan**<br><br>*Are fundraising activities to mobilize resources being implemented?*<br>*Are fundraising activities included in the annual plan?*	There are no fundraising activities in the community to mobilize resources. There is no mention of fundraising activities in the annual plan (if there is an annual plan)	Fundraising activities are ad hoc. Discussion have been held about including fundraising activities in the annual plan and conversations have been held in the community about	Fundraising activities to mobilize resources occur occasionally. Fundraising is purposeful-an activity is identified for which the funds will be use (i.e., environmental sanitation, etc.). There is at least one fundraising activity included in the annual plan	Fundraising activities occur routinely and several targets have been established. Several fundraising activities are included in the annual plan. Several activities have been identified for use of the funds raised with some committee i[illegible]volvement. [illegible]ere is some [illegible]versification of sources and methods for fundraising	Fundraising is a priority, occurs frequently, and targets have been set for all fundraising activities. Several fundraising activities are included in the annual plan and involve diverse sources and methods. All fundraising activities have been associated with a specific activity and with full committee involvement
**3.2 Financial (or other assets) Documentation and Transparency**<br><br>*Are proper financial records kept and shared with the committee and the community?*	There are no financial/asset records kept. Financial/asset updates are not shared with full committee	There are some financial/asset records kept but proper bookkeeping methods are not used. Financial/asset records are not easily accessible to committee members and rarely shared at meetings	Financial/asset records are being kept using proper bookkeeping methods. Financial/asset records are shared regularly at meetings. Financial/asset records are not shared with the community	Financial/asset records are kept using proper bookkeeping methods and analyzed using basic tools. A regular schedule of updates/review (quarterly balance, semi-annual budget review, annual report) is attempted. Financial information is rarely shared with the community. There is a discussion during a committee meeting about opening a bank account	Detailed financial records are being kept and being analyzed using more advanced tools/method. The regular schedule of updates/reviews is mostly followed. The annual financial report is shared with the community. A bank account has been opened and it used to manage funds

Indicator	1st Stage 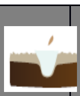	2nd Stage 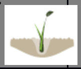	3rd Stage 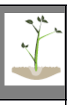	4th Stage 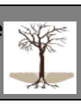	Final Stage 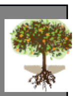
**3.3 Other Resource Mobilization**<br><br>*Are HMC/WDC members aware of local resources and utilize them to implement activities?*	HMC/WDC does not have a regular meeting place within the community. HMC/WDC members do not utilize local resources to implement activities	HMC/WDC has a temporary meeting place in the community. Utilizing local resources to implement activities is rarely	The HMC/WDC has a permanent meeting space but it is not a convenient space (i.e. bad location, too small, etc.). Local resources are occasionally utilized to implement activities	The HMC/WDC has a proper permanent meeting space. Members are very familiar with local resources available. Local resources are often utilized but documentation of resources is poor	HMC/WDC has an established meeting place in a central location that is well known throughout the community .Utilizing local resources is a habit (routine practice) and a list of available resources is created and updated annually
**3.4 Mobilize residents of the ward to implement self-help, communal/voluntary, and/or development projects**<br><br>*Are HMC/WDC members mobilizing and organizing the community members to participate in community activities that are voluntary and contribute to helping the residents or improve the standards in their community?*	HMC/WDC members do not mobilize the community for voluntary or development activities. There is no evidence in the annual plan of mobilizing the community for voluntary activities	HMC/WDC members mobilize the community occasionally when prompted by other actors such as an NGO or the DHMT, FCC. HMC/WDC require full support from an external source to organize and finance the activity	HMC/WDC members demonstrate initiative and mobilize the community at least once per year, sometimes requiring the support of an external actor. HMC/ WDC show some initiative and occasionally mobilize the community but it is not planned in advance and the community is not notified until the day of the activity. Organization of the activity is ad hoc and there is little or no collaboration with other actors such as the health facility, FCC, DHMT or NGOs	HMC/WDC members demonstrate initiative and do not require external support to mobilize and implement an activity in the community at least twice per year. Mobilization is usually planned according to the annual plan and the community is notified at least three days in advance of the activity There is a representative from the HMC/WDC appointed to lead on the activity and	HMC/WDC members demonstrate initiative and do not require external support to mobilize and implement an activity in the community 3- 4 times per year. Mobilization is planned according to the annual plan and the community is notified at least seven days in advance of the mobilization and implementation of the activity. Other key stakeholders are involved in the mobilization and implementation of the activities. Key stakeholders

Indicator	1st Stage 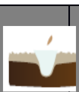	2nd Stage 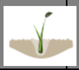	3rd Stage 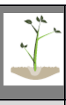	4th Stage 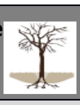	Final Stage 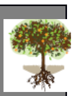
				there is evidence of stakeholder engagement	have specific roles or have made contributions to the activities

Capacity Area IV: Collaboration and Coordination

Definition: The WDC/HMCs ability to establish relationships with key community, district, and relation institutions, resulting in an increase of services in support of the community.

Indicator	1st Stage 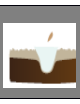	2nd Stage 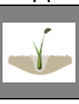	3rd Stage 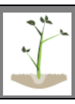	4th Stage 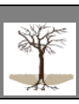
**4.1 Collaboration and Coordination with other WDC/HMCs**<br>*Does the WDC/HMC collaborate and coordinate with other WDC/HMCs?*	WDC/HMC has no communication with other WDC/HMCs.	WDC/HMC realizes the benefit of establishing relationships with other WDC/HMCs.<br>WDC/HMC has taken some steps towards collaborating with other WDC/HMCs such as initiating contact or setting-up a preliminary meeting.	WDC/HMC has had 1-2 meetings with other WDC/HMCs to share lessons learned and coordinate activities.	WDC/HMC is in regular contact with 1-2 other WDC/HMCs. Some meetings are held to discuss issues an[illegible] share learning. The process of starting an annual meetin[illegible] between all WDC/HMCs in the municipality has begun.
**4.2 Collaboration and Coordination with health facilities, DHMT, and FCC**<br><br>*Does the WDC/HMC collaborate and coordinate with health facilities, DHMT, and the FCC*	WDC/HMC has no established relationship with the PHU/health facility. WDC/HMC has no established relationship with the DHMT and/or FCC	WDC/HMC has made contact with the PHU and PHU staff know the function of the WDC/HMC. WDC/HMC has made contact with the DHMT and/or FCC. Collaboration is rare and infrequent	WDC/HMC has established a formal relationship with the PHU and they meet on an ad hoc basis. WDC/HMC collaborates with DHMT and FCC for special occasions only. Collaboration is organized and regular, either through scheduled meetings or routine visits	WDC/HMCs relationship wit[illegible] the PHU has become institutionalized and they meet at least quarterly. WDC/HMC collaborates wit[illegible] the DHMT and FCC on short and long-term initiatives an[illegible] continuously seeks further opportunities for collaboration.

Capacity Area IV: Collaboration and Coordination

Indicator	1st Stage 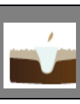	2nd Stage 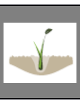	3rd Stage 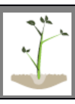	4th Stage 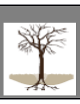
**4.3 WDC/HMC Support to CHW Peer Supervisors and CHWs**<br><br>*Does the WDC/HMC support CHW Peer Supervisors and CHWs in their work?*	WDC/HMC do not engage with CHW Peer Supervisors and there is no representation of CHW Peer Supervisors in WDC/HMC meetings.<br>There is no collaboration with CHWs in the community regarding WDC/HMC activities. The WDC/HMC does not offer or demonstrate support to CHW Peer Supervisors and CHWs in their work	CHW Peer Supervisor representatives occasionally participate in discussions and share the views of CHWs and their colleagues with WDC/HMCs.<br>WDC/HMC rarely support CHWs and CHW Peer Supervisors in their work. Support would be provided only as requested by CHW Peer Supervisors on special occasions	WDC/HMC invite CHW Peer Supervisor Representative to meetings, engage/collaborate with them on community mobilization activities and make some effort to inform CHW Peer Supervisors regarding WDC/HMC activities in the community.<br>WDC/HMC sometimes supports CHW Supervisors by helping with collection of information or overseeing CHW activities	WDC/HMC regularly liaise with CHW Peer Supervisors in the community regarding CHW activities.<br>The WDC/HMC have taken preliminary steps to establis system to help CHW Peer Supervisor with activities suc as identifying beneficiaries and/or vulnerable householc collecting health informatior or household level data, and supervising CHW Peer Supervisors in the field

Capacity Area V: ***Monitoring and Evaluation***

Definition: The WDC/HMC's ability to systematically document the results of its activities and ensure this information is regul basis for future planning. The WDC/HMC actively supports the collection of community health data and uses relevant informa process.

**Indicator**	**1st Stage** 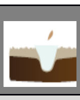	**2nd Stage** 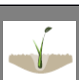	**3rd Stage** 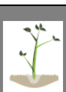	**4th Stage** 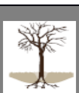	**Final Stage** 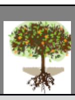
**5.1 Review of Annual Plan**<br><br>*Is the annual plan regularly followed and reviewed at the end of every year?*<br><br>*Are annual review results used in creating the next year's annual plan and long term plans?*	There is no annual review. There is no yearend review. Annual review results and recommendations do not exist or are not referred to in planning or other decisions.	The HMC/WDC does not look at the annual plan throughout the year to check its progress. At the end of the year, the HMC/WDC holds a meeting to review the year's accomplishments but the results are not written. Annual review results and recommendations are rarely considered in creating new annual plan and in planning throughout the year	The HMC/WDC rarely looks at the annual plan to check its progress and for further planning. A year-end review meeting is held and the results are recorded. There is an attempt to gather information on all activities conducted prior to the year-end review meeting. HMC/WDC prepare a basic evaluation report and share with the health facility, DHMT, FCC and NGOs. Some annual review results and recommendations are considered in creating a new annual plan and in planning throughout the year.	The HMC/WDC looks at the annual plan to check its progress and for further planning a few times a year but not at set intervals. Quarterly review meetings are held and the results are recorded. An evaluation report including quantified results for most activities conducted is prepared and shared with the health facility, DHMT, FCC and NGOs.<br>Most annual review results and recommendations are considered in creating a new annual plan and in planning throughout the year	The HMC/WDC looks at the annual plan quarterly to check its progress and for further planning.<br>Information on all activities conducted are gathered and summarized at the quarterly review meeting. A comprehensive evaluation report is prepared and shared with the community, health facility, DHMT, FCC, and NGOs yearly. A system is in place to ensure all annual review results and recommendations are considered in creating a new annual plan and in planning throughout the year
**5.2 WDC/HMC Use Health Information in Planning**<br><br>*Does the WDC/HMC ensure data quality control in health data collection and consider health information in planning?*	WDC/HMC does not receive or consider health information during planning of community activities. There is no shared understanding of the value of community level surveillance. HMC/WDC are not involved in the data collection, supervision or aggregation of household level	Community or district health information is available but rarely considered by the HMC/WDC during planning of community activities. HMC/WCDs acknowledge the importance and value of community level health data. HMC/WDCs realize one of their responsibilities is to manage the data	Community and district health information is sometimes considered by HMC/WDC during monthly and quarterly planning of community health activities. There is some effort to ensure timely reporting and quality of some health data from the CHW Peer Supervisors reports but it is not done on a regular basis. Reports are not always complete	Community and district health information is regularly considered by HMC/WDC during monthly and quarterly planning of activities. Steps are regularly taken to ensure that high-quality community health information is collected and consolidated on a monthly basis. HMC/WDC members review the quality and completeness of most of the CWH	Community and district health information is always considered by HMC/WDC during planning and information is included in quarterly and annual plans. Community data is reviewed and discussed at all monthly meetings. There is evidence that decisions are made base on community health data. A quality control system is in place and steps

Indicator	1st Stage 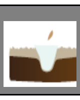	2nd Stage 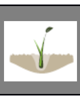	3rd Stage 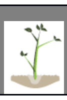	4th Stage 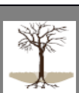	Final Stage 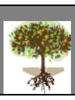
	data. No system is in place to ensure that community level health data is collected and submitted. HMC/WDC do not feel accountable for health data	collection process, particularly the consolidation of CHW Peer Supervisors reports. Reminders/advice are sometimes given to CHW Peer Supervisors regarding quality control for health data but no steps are taken to check quality	and the data is several months old. Reminders/advice are sometimes given to CHW Supervisors regarding quality control for health data and some steps are taken to check quality	Peer Supervisors reports. Discussions are held with PHU staff and CHW Peer Supervisors about the community data during some meetings. Feedback is provided to PHU staff and CHW Peer Supervisors about the quality of the data and discussions are held routinely on what course of action needs to be taken	have been taken to ensure that vital community health events (births, deaths) are investigated and recorded. HMC/WDC members review the quality and completeness of all of the CWH Supervisors reports and feedback is given to each supervisor on a monthly basis. Information is fed up to the DHMT and FCC. Discussions are held with PHU Staff and CHW Peer Supervisors about the community data during all meetings. Action points are documented and reviewed at the next meeting

Capacity Area VI: Supervision

Definition: The WDC/HMC routinely and systematically supervise CHW Peer Supervisors and provide timely feedback on
WHC/HMCs are involved in and oversee the CHW training and activity plans and ensure these activities are in accordanc
and respond to the needs of the community. The WDC/HMC are the liaison between the CHW Supervisors and PHU Staf

Indicator	1st Stage 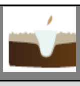	2nd Stage 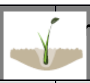	rd Stage 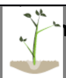	h Stage 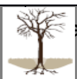	al Stage 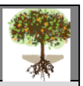
**6.1 Regular and Systematic supervision of CHW Peer Supervisors** *Are CHW Supervisors being supervised by HMC/WDC on a quarterly basis? Is there evidence of a supervision schedule to ensure all supervisors receive a visit once per quarter? Is performance feedback being given to supervisors and action points developed for areas which need improvement*	There is no evidence of planned or routine supervision by HMC/WDC members for the CHW Supervisors	Supervision has been discussed during HMC/WDC meetings .A schedule or plan has been developed to ensure each CHW Supervisor receives a visit quarterly. A performance feedback checklist/form has been developed and HMC/WDC members have been trained on supervision and how to provide feedback	-Supervision is occurring periodically but not every CHW supervisor has received a visit from a HMC/WDC member at least once per quarter. Supervision is ad hoc and the schedule is not strictly adhered to. Performance feedback forms are being implement but not routinely used or are not fully completed	Supervision is occurring routinely and most CHW supervisors receive at least once supervision visit by a HMC/WDC member once per quarter. A supervision schedule is routinely used by all members and discussed during monthly meetings. Performance feedback forms are being used correctly and mostly complete. All CHW Supervisors have received two performance feedback forms from their HMC/WDC representative	Supervision is occurring according to the schedule and each CHW Supervisor has received at least one visit by an HMC/WDC member once per quarter. Performance feedback forms are used correctly and consistently; all forms are complete. Action points have been developed by HMC/WDC members based on CHW Supervisors performance and these are reviewed during monthly meetings and reviewed during quarter performance feedback visits with the supervisors
**6.2 Monthly and quarterly meetings in which supervision is an agenda item**	During HMC/WDC monthly meetings with PHU staff and CHW Representatives, supervision is never discussed and performance feedback is not being practiced	HMC/WDC hold monthly meetings with PHU staff and CHW representatives and occasionally supervision is included in the	Supervision is included in the agenda at least every other meeting and sometimes performance feedback forms are	Supervision is a routine topic in HMC/WDC meeting. Performance feedback forms are reviewed, issues are discussed and	Supervision is an agenda item at every HMC/WDC meeting<br>Action points developed from prior meetings on performance and supervision are reviewed and areas of

Indicator	1st Stage	2nd Stage	[illegible]rd Stage	[illegible] Stage	[illegible]l Stage
		agenda. There is no mention of the performance feedback	reviewed and issues are discussed	actions points are developed. CHW Supervisors are invited to contribute to the dialogue solutions	underperformance are discussed and a strict course of action is developed. CHW Supervisors participate in action planning to resolve issues in performance and supervision
**6.3 HMC/WDC review and contribute to CHW activity plans**	HMC/WDC do not participate in planning activities with CHWs and CHW Supervisors	HMC/WDC are aware of the CHW activities however HMC/WDCs have not reviewed the activities in advance or advised CHWs and CHW Supervisors of the activities planned according to the HMC/WDC annual plan	HMC/WDCs have review CHW activity plans however these plans were not developed in collaboration with the HMC/WDC and there is no evidence that the annual plan was consulted when the CHW activity plans were developed	HMC/WDC contributed to the development of the CHW activity plans by providing feedback to CHW Supervisors. CHW Supervisors were provided with a copy of the HMC/WDC annual plan to inform the development of CHW activities	HMC/WDC participated in the development of CHW activity plans and these were presented during monthly meeting and agreed to by all in attendance. CHW activity plans have been signed off by the Chairman of the HMC/WDC .Activities are in line with the annual plan

Capacity Area VI: Supervision

HICAP Score Card Booklet

Name of Community:

____

Capacity Area:

Capacity Area	Baseline Score	Assessment 1	Assessment 2	Assessment 3	Endline Score:
Capacity Area I: Participatory Planning					
Capacity Area II: Leadership (Governance)					
Capacity Area III: Resource Mobilization & Management					
Capacity Area IV: Collaboration & Coordination					
Capacity Area V: Monitoring and Evaluation					
Capacity Area VI: Supervision					

How to Score the Capacity Areas:

The five-stage scale represented through images of seed development demonstrates the growth/progression for each capacity area and the corresponding sub-indicators. The stages start at seed sowing (1), to germination (2), then sapling (3), followed by maturing (4), and finally flowering rearing fruit (5).

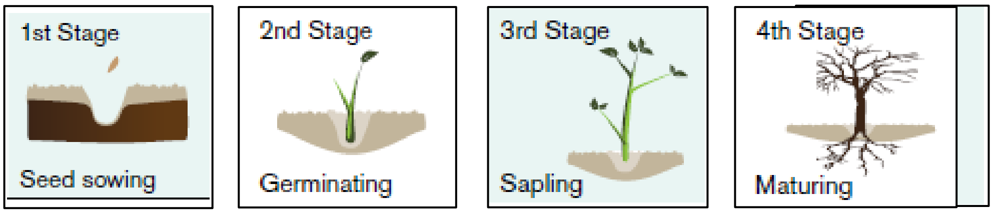

<u>Stage 1 =Score of 1</u>
Seed sowing: Elementary/beginning stage of capacity that implies the start of any project; no results are visible yet the origin exists.

<u>Stage 2= Score of 2</u>
Germination: The input has produced a small result, which may grow into something bigger if the necessary care continues to be given. The second stage indicates there is a possibility of achieving the future dream.

<u>Stage 3= Score of 3</u>
Sapling: Through significant efforts over the time, the possibility of reaching the dream becomes more and more viable as the probability of the intended result has sharply increased and becomes less vulnerable to destructive external factors.

<u>Stage 4= Score of 4</u>
Maturing: A series of mechanism of inputs results in increased strength and the likelihood of the eventual dream. There is less of a significant threat for sustainability, and all of the efforts and mechanism that contribute to the growth become well-functioning and viable.

<u>Stage 5= Score of 5</u>
Fruit Bearing: The dream is realized and the permanent changes and results have taken place. Caregivers and people fully benefit from the results, and the outcome provides continuous benefits. More importantly, there is very little possibility of the outcome ending.

Check the box for corresponding assessment:

Baseline ☐ **Assessment 1** ☐ **Assessment 2** ☐ **Assessment 3** ☐ **Endline** ☐

Date of Assessment:

Capacity Area I: Participatory Planning
The systems in place to ensure HMC/WDC activities are planned in advance, with proper division of responsibilities, phases of implementation, and input from all HMC/WDC members.

1.1 Meeting Attendance	**Score:**
1.2 Regular Meetings with an agenda and an elected chair to lead the meeting	**Score:**
1.3 Written Quarterly or Annual Plan	**Score:**

Average Score Capacity Area 1 : ________

[example calculation for average : (score for 1.1 + score for 1.2 + score for 1.3)/ (total number of indicators for capacity area 1); for example, if indicator 1.1 received a score of 3, 1.2 received a score of 2, and 1.3 received a score of 4 then: (3+2+4)/3= 3]

Previous Score Capacity Area 1:

____

Target Score Capacity Area 1: __________

→Review goals from prior assessment

- How many goals were developed? ______
- How many goals were accomplished? ______

Goals for next assessment for Capacity Area I: Participatory Planning
Person(s) Responsible for goal:

**Capacity Area II: Leadership (Governance)**<br>The processes followed to ensure the HMC/WDO remains representative of and responsible to the community, through proper internal management ensuring all members understand their responsibilities, good character, and fully participate in decision making to achieve a common goal.	
2.1 Membership Election and Replacement Process	**Score:**
2.2 Committee roles assigned and understood	**Score:**
2.3 Demonstrated leadership capacity	**Score:**
2.4 Participatory Decision Making	**Score:**
2.5 There is a 50/50 gender balance on the committee	**Score:**
2.6 Community Knowledge and Perception of WDC/HMC	**Score:**

Average Score Capacity Area 2: __________

[example calculation for average : (scores of 2.1+ scores of 2.2 + scores of 2.3 + scores of 2.4 + scores of 2.5 +scores of 2.6)/ (total number of indicators in capacity area 2: 6)]

Previous Score Capacity Area 2:

____

Target Score Capacity Area 2: __________

→Review goals from prior assessment

- How many goals were developed? _____
- How many goals were accomplished? _____

Goals for next assessment for Capacity Area II: Leadership (Governance)

Person(s) Responsible to goal:

**Capacity Area III: Resource Mobilization and Management**<br>The HMC/WDC ability to raise funds, locate and utilize resources and maintain proper financial records available to the public.	
3.1 Fundraising activities are implemented in the community and included in the Annual Plan	**Score:**
3.2 Financial (or other assets) Documentation and Transparency	**Score:**
3.3 Other Resource Mobilization	**Score:**
3.4 Mobilize residents of the ward to implement self-help, communal/voluntary, and/or development projects	**Score:**
Average Score Capacity Area 3:<br>*Previous Score Capacity Area 3:* __________<br>*Target Score Capacity Area 3:* __________<br>→Review goals from prior assessment<br>○ How many goals were developed? _____<br>○ How many goals were accomplished? _____<br>Goals for next assessment for Capacity Area III: Resource Mobilization and Management<br>Person(s) Responsible to goal:	
**Capacity Area IV: Collaboration and Coordination**<br>The WDC/HMCs ability to establish relationships with key community, district, and relation institutions, resulting in an increase of services in support of the community.	
4.1 Collaboration and Coordination with WDC/HMCs	**Score:**
4.2 Collaboration and Coordination with health facilities, DHMT, and FCC	**Score:**
4.3 WDC/HMC Support to CHW Peer Supervisors and CHWs	**Score:**

Average Score Capacity Area 4:

Previous Score Capacity Area 4:

____

Target Score Capacity Area 4:   __________

→Review goals from prior assessment

- How many goals were developed? _____
- How many goals were accomplished? _____

Goals for next assessment for Capacity Area IV: Collaboration and Coordination
Person(s) Responsible to goal:

Capacity Area V: Monitoring and Evaluation

The WDC/HMC's ability to systematically document the results of its activities and ensure this information is regularly reviewed and used as the basis for future planning. The WDC/HMC actively supports the collection of community health data and uses relevant information to inform its planning process.

5.1 Review of Annual Plan	**Score:**
5.2 WDC/HMC use health information in planning	**Score:**

Score Capacity Area 5:

Previous Score Capacity Area 5:
____

Target Score Capacity Area 5: __________

→Review goals from prior assessment

- How many goals were developed? _____
- How many goals were accomplished? _____

Goals for next assessment for Capacity Area V: Monitoring and Evaluation
Person(s) Responsible to goal:

**Capacity Area VI: Supervision**<br>The WDC/HMC routinely and systematically supervise CHW Peer Supervisors and provide timely feedback on their performance. The WHC/HMCs are involved in and oversee the CHW training and activity plans and ensure these activities are in accordance with the HMC/WDC annual plans and respond to the needs of the community. The WDC/HMC are the liaison between the CHW Supervisors and PHU Staff.	
6.1 Regular and Systematic supervision of CHW Peer Supervisors	**Score:**
6.2 Monthly and quarterly meetings in which supervision is an agenda item	**Score:**
6.3 HMC/WDC review and contribute to CHW	

Average Score Capacity Area 6:

Previous Score Capacity Area 6: __________

Target Score Capacity Area 6: __________

→Review goals from prior assessment

- How many goals were developed? _____
- How many goals were accomplished? _____

Goals for next assessment for Capacity Area VI: Supervision
Person(s) Responsible to goal:

Signatures of all attendees:
Date for next assessment: __ / __ / ____

1.
2.
3.
4.
5.
6.
7.
8.
9.
10.
11.
12.
13.
14.
15.
